# Multilayered analysis of cisplatin resistance mechanisms in bladder cancer: from the cell membrane to organelles

**DOI:** 10.7717/peerj.21148

**Published:** 2026-04-24

**Authors:** Longtu Ma, Yan Tao, Long Cheng, Chengyu You, Xi Xiao, Weiguang Yang, Yidong Liu, Zhilong Dong

**Affiliations:** 1 Department of Urology, The Second Hospital & Clinical Medical School, Lanzhou University, Lanzhou, China; 2 Institute of Urology, Clinical Research Center for Urology in Gansu Province, Key Laboratory of Urological Disease in Gansu Province, Lanzhou University Second Hospital, Lanzhou, China; 3 The Affiliated Taian City Central Hospital of Qingdao University, Taian, China

**Keywords:** Cisplatin, Resistance, Molecular mechanisms, Bladder cancer

## Abstract

Bladder cancer (BCa) is one of the most common malignancies of the urinary tract worldwide. Cisplatin-based combination chemotherapy remains a cornerstone of treatment for muscle-invasive and advanced disease and has substantially improved clinical outcomes, yet primary and acquired resistance frequently leads to treatment failure and disease recurrence. Classical mechanisms, including altered drug uptake and efflux and detoxification by glutathione or metallothioneins, account for only part of this phenotype. Recent work in BCa increasingly points to cisplatin resistance as a multilayered cellular adaptation involving coordinated changes in drug handling, stress responses, and cell-death control. Drawing primarily on studies published between January 2020 and April 2025, while incorporating selected foundational studies from the preceding decade, this review maps cisplatin resistance in BCa within a structured “cell membrane and tumor microenvironment–cytoplasm–nucleus and chromatin–organelles” framework. Particular emphasis is placed on the interaction of epithelial–mesenchymal transition and cancer stem cell programs with stromal and immune signals at the membrane level; on metabolic rewiring, ferroptosis regulation, and stress-activated signaling cascades in the cytoplasm; on reinforced DNA damage response pathways and RNA- or chromatin-directed epigenetic remodeling in the nucleus; and on the resetting of apoptotic, autophagic, and mitophagic thresholds at the organelle level. Across these compartments, recurrent regulatory nodes and signaling axes are outlined, and areas are delineated where mechanisms are supported by convergent *in vitro*, *in vivo*, and clinical evidence versus those that remain primarily exploratory. By viewing cisplatin resistance in BCa as an integrated and dynamic network spanning cellular compartments, this multilayered synthesis aims to refine current mechanistic concepts and to provide a rationale for biomarker development and combination strategies designed to prevent or overcome cisplatin resistance.

## Introduction

Bladder cancer (BCa) is the tenth most common malignancy worldwide, with approximately 573,000 new cases and 213,000 deaths reported annually. Approximately 75% of patients present with non–muscle-invasive bladder cancer (NMIBC), whereas the remainder are diagnosed with muscle-invasive disease (MIBC) (Sung et al., 2021). Patients with MIBC generally have poor prognoses. Although intravesical Bacillus Calmette–Guérin (BCG) instillation is widely used to reduce recurrence and progression in high-risk NMIBC, the lack of robust molecular biomarkers limits precise prediction of treatment response and timely intervention (Pedersen et al., 2023). Radical cystectomy remains the standard treatment for localized MIBC (Li et al., 2022), yet the five-year survival rate is only 20–40%, and nearly half of patients develop metastatic disease within three years.

Against this background, systemic therapy has become a crucial component of BCa management. Cisplatin-based combination chemotherapy has been widely used since the late 1980s and has been established as the first-line regimen for metastatic BCa (Ghandour, Singla & Lotan, 2019). Although newer platinum analogues have been developed to reduce toxicity, cisplatin remains the backbone of both perioperative and palliative regimens (Galanski, 2006; Connors et al., 2023). However, only about 35% of patients with metastatic BCa achieve an initial objective response to cisplatin-based chemotherapy, and most responders ultimately experience disease progression due to acquired resistance (Fotopoulou, Titilas & Ronconi, 2022). Classical mechanisms, including altered drug uptake and efflux, increased intracellular sequestration and detoxification *via* glutathione and metallothioneins, and enhanced DNA repair, account for only part of this resistant phenotype. Increasing evidence suggests that cisplatin resistance in BCa reflects a multilayered adaptive state involving coordinated changes in drug handling, cellular stress responses, and cell-death regulation.

**Figure 1:**
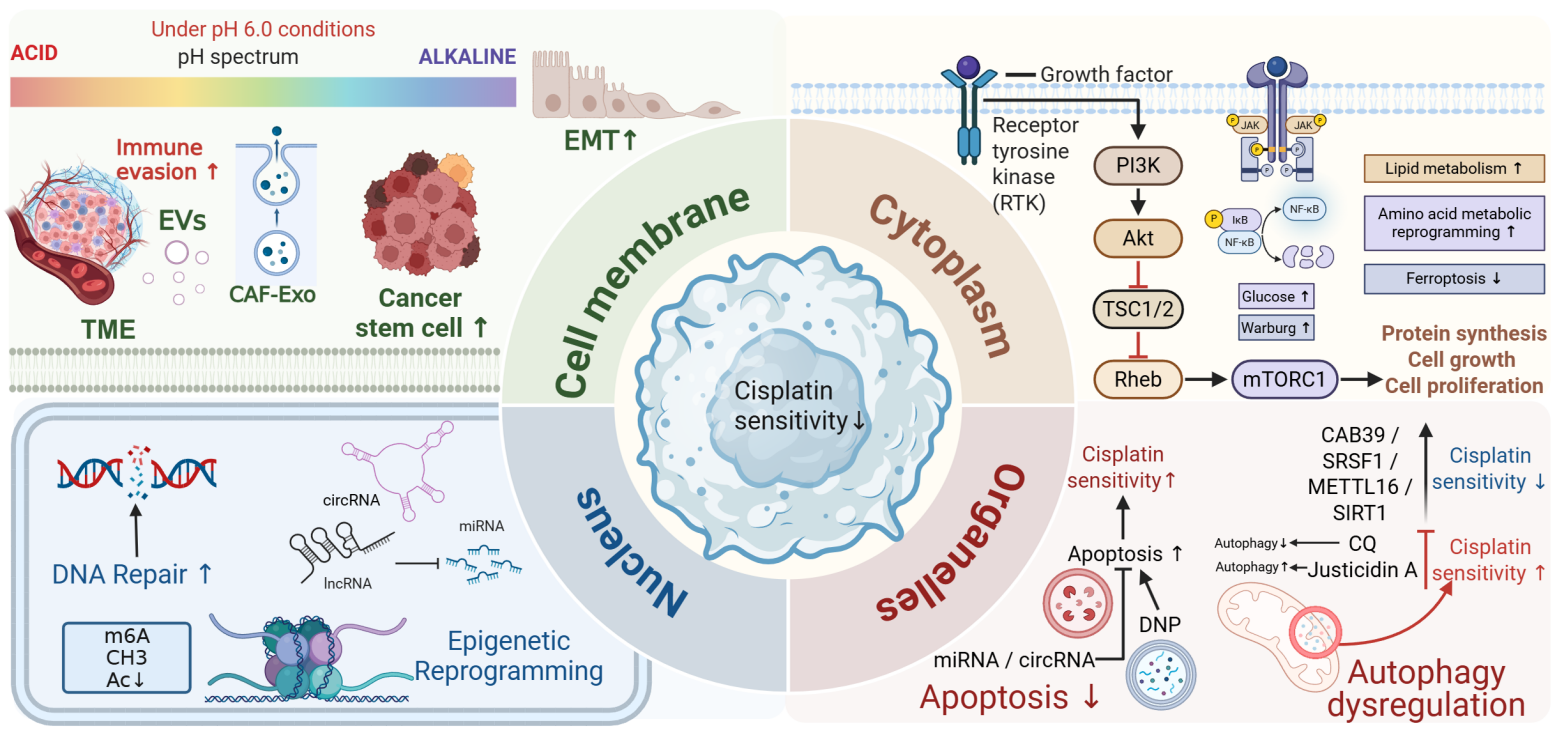
Integrated network model of cisplatin resistance in bladder cancer. Integration of the reviewed mechanisms into four interacting compartments: at the cell membrane/TME level (upper left), EMT/CSC expansion, acidic pH and CAF/immune cell–derived EVs reshape drug influx–efflux balance, while cytoplasmic signalling (upper right; PI3K–Akt–mTOR, JAK/STAT, NF-κB) rewires glycolysis, lipid/amino-acid metabolism and ferroptosis control. In the nuclear quadrant (lower left), enhanced DNA repair and epigenetic reprogramming (m^6^A/DNA/histone and ncRNA circuits) stabilise pro-survival transcription, whereas the organelle quadrant (lower right) highlights how mitochondrial apoptosis and (dys)regulated autophagy/mitophagy, modulated by molecules such as CAB39, SRSF1, METTL16 and SIRT1, dynamically tune cisplatin sensitivity (↑/↓ arrows). Figure created with BioRender.com.

In light of these developments, there is a need for a BCa-focused synthesis that brings together compartment-specific mechanisms into a coherent framework. In this review, we focus primarily on studies published between January 2020 and April 2025, while incorporating selected foundational studies from the preceding decade, and map cisplatin resistance within a structured “cell membrane and tumor microenvironment–cytoplasm–nucleus and chromatin–organelles” framework. Within this structure, we summarize how epithelial–mesenchymal transition and cancer stem cell programs interface with stromal and immune cues at the membrane level; how metabolic rewiring, ferroptosis control, and stress-activated signaling cascades shape cytoplasmic adaptation; how DNA damage response pathways and RNA- or chromatin-directed epigenetic regulation contribute at the nuclear level; and how apoptosis, autophagy, and mitophagy are reorganized at the level of organelles. Across these compartments, several recurrent regulatory nodes emerge, including DNA damage response–epigenetic hubs, glutathione and mevalonate metabolism, ferroptosis-modulating circuits, and exosome-mediated noncoding RNA networks, which appear to coordinate cross-layer adaptation and therefore represent plausible points for therapeutic co-targeting with cisplatin or immune checkpoint inhibitors. These considerations converge on a central question: in molecularly defined subsets of BCa, can durable resensitization to cisplatin be achieved by concomitantly modulating key nodes distributed across more than one cellular compartment? Addressing this question will require study designs that explicitly account for pronounced intratumoral heterogeneity, the limited availability of well-annotated cisplatin-treated cohorts, and the current lack of robust, standardized assays that can be implemented in routine diagnostic workflows. An overview of the multilayered mechanisms of cisplatin resistance in BCa is presented in Fig. 1.

## Survey methodology

Search strategy and study selection. We searched PubMed, Web of Science, and Google Scholar for peer-reviewed articles on bladder cancer cisplatin resistance using predefined terms (*e.g.*, “cisplatin resistance”, “bladder cancer”, “EMT”, “DNA repair”, “autophagy”, “ferroptosis”, “signaling pathway”, “ROS”, and “NHEJ/HRR”). The primary search window covered studies published between January 2020 and April 2025, while selected seminal studies from the preceding decade were included when foundational to current understanding. We included original research and mechanistic studies (cell, animal, multi-omics, digital pathology/artificial intelligence) and narrative or scoping reviews that synthesized mechanisms. We excluded case reports, non-English items, and sources without primary or integrative evidence. Two authors independently screened titles and abstracts, reviewed full texts for relevance, and resolved disagreements by consensus. Reference lists of key papers were snowballed to minimize omissions. Our goal was to produce a comprehensive yet unbiased synthesis of multilayered mechanisms. When multiple studies addressed the same node or pathway, we prioritized methodological rigor, validation (*e.g.*, external cohorts), and mechanistic coherence.

## Cell membrane

### EMT (epithelial–mesenchymal transition) and the acquisition of cancer stemness traits

Epithelial–mesenchymal transition (EMT) describes a dynamic state in which epithelial cells gradually lose polarity and cell–cell adhesion while acquiring a more motile, mesenchymal-like phenotype. Canonical features include reduced E-cadherin and increased Vimentin and N-cadherin, together with induction of SNAIL, TWIST, and ZEB family transcription factors (Lai et al., 2020). In bladder cancer, EMT-like changes typically coincide with enhanced migration, invasion, and broad stress tolerance and are frequently accompanied by decreased sensitivity to cytotoxic chemotherapy, including cisplatin (Lima De Oliveira et al., 2023). EMT is also tightly connected to the emergence of cancer stem cell (CSC) traits: cells in partial EMT states tend to display self-renewal, multipotent differentiation, and survival advantages under drug pressure, providing a cellular reservoir from which cisplatin-resistant subclones can arise (Zhang et al., 2012). Importantly, most of these conclusions are derived from *in vitro* systems and retrospective tissue analyses, and EMT in patients likely exists along a spectrum of hybrid states rather than as a simple binary switch, which complicates its direct use as a predictive biomarker.

Several studies have begun to dissect how noncoding RNAs and protein regulators converge on this EMT–CSC axis, but the depth and quality of evidence vary considerably across pathways. circATIC, the OCT4-pg5/miR-145-5p/OCT4B network, and hypoxia-induced HOXA-AS3 have each been reported to promote EMT and blunt cisplatin-induced cell death by functioning as competing endogenous RNAs and activating Wnt/β-catenin or Notch1 signaling. In these models, circATIC and OCT4-pg5 correlate with more aggressive clinicopathological features, whereas HOXA-AS3 knockdown combined with cisplatin suppresses tumor growth in xenografts, providing at least some *in vivo* support for this lncRNA–Notch1 axis (Huang et al., 2024a; Zhou et al., 2024; Chen et al., 2020). Nonetheless, these studies are largely based on a limited number of urothelial carcinoma cell lines, often without validation across molecular subtypes or independent clinical cohorts, so whether these ncRNA circuits represent universal drivers or context-dependent modifiers of resistance remains unresolved. Protein regulators such as ADNP and PRDX2 have also been implicated: both promote EMT and chemoresistance by engaging TGF-β/Smad and PI3K–AKT–mTOR signaling and are associated with tumor progression and poor outcome in patient series, suggesting stronger translational relevance than purely cell-based observations (Xie et al., 2021; Li et al., 2024c). Within this group, TYMP stands out. As part of a CEBPB/TYMP/GDF15 axis, it drives EMT, tumor growth, and cisplatin resistance in multiple cell lines and mouse models, and pharmacological inhibition with TAS-102 significantly enhances cisplatin cytotoxicity *in vivo*, pointing to a potentially druggable node (Tian et al., 2025). Even here, however, prospective clinical evidence is lacking, and on-target toxicity in normal tissues that depend on the same stress-response pathways has yet to be systematically evaluated.

CSC-like subpopulations form a second major pillar of this module and are closely interwoven with EMT. Bladder CSCs show self-renewal capacity, multipotent differentiation, high activity of drug-efflux pumps, reinforced DNA damage repair, and strong anti-apoptotic signaling, features that together confer intrinsic tolerance to cisplatin (Chen & Chang, 2019). Interactions with the tumor microenvironment further amplify these traits: CAF-derived exosomes enriched in miR-146a-5p strengthen CAF–tumor cell contacts, activate STAT3 and mTOR signaling by targeting ARID1A and PRKAA2, and upregulate the ECM protein SVEP1, thereby enhancing CSC properties and resistance to gemcitabine–cisplatin, while circulating exosomal miR-146a-5p levels correlate with stage and recurrence risk and may serve as a noninvasive marker of aggressive, drug-tolerant disease (Zhuang et al., 2023). At present, however, the clinical series used are relatively small and often single-center, so their predictive value still needs confirmation in larger, treatment-stratified cohorts. At the metabolic level, the circFOXK2–HSP90β–TACO1 complex promotes mitochondrial complex IV activity, oxidative phosphorylation, and mtROS, supporting CSC maintenance and cisplatin resistance; increased mitochondrial localization of TACO1 is associated with poor response to platinum-based chemotherapy in patient samples, which strengthens the link between this pathway and clinical resistance (Deng et al., 2025a). Pharmacological targeting of stemness-linked transcriptional regulators, such as CDK7 inhibition with the covalent inhibitor THZ1, reduces sphere formation, induces cell death in both chemo-naive and chemo-resistant urothelial carcinoma cell lines, and suppresses xenograft growth, highlighting CSC-directed transcriptional control as a promising, though still preclinical, therapeutic entry point (Chow et al., 2021). In contrast, data on TRPV4 are at an early stage: TRPV4 is upregulated in bladder cancer tissues, increases Ca2+ influx, and promotes proliferation and migration *in vitro*, and its expression is downregulated by cisplatin in sensitive but not resistant cells, suggesting a possible contribution of Ca2+ signaling to the EMT–CSC network. However, direct causal evidence that TRPV4 drives cisplatin resistance *in vivo* is currently limited, and its role should for now be regarded as hypothesis-generating rather than firmly established (Katari et al., 2025). Overall, available evidence supports a model in which EMT and CSC phenotypes are sustained by interconnected ncRNA, transcriptional, signaling, and metabolic circuits. Among these, pathways that have been validated across cell lines, animal models, and clinical cohorts, such as HOXA-AS3/Notch1, CEBPB–TYMP–GDF15, and circFOXK2–HSP90β–TACO1, appear more robust, while many individual ceRNA networks remain preliminary observations that require broader and more rigorous evaluation.

### Remodeling of the tumour microenvironment (TME)

The tumor microenvironment (TME) makes an indispensable contribution to cisplatin resistance in BCa. Beyond genetic and epigenetic alterations within cancer cells, fibroblasts, immune cells, extracellular vesicles (EVs), and local physicochemical conditions cooperate to create a niche that supports survival under chemotherapy. Through paracrine signaling and metabolic crosstalk, this niche can sustain EMT/CSC programs, enhance DNA repair, and blunt cell-death pathways, gradually shifting the balance toward a drug-tolerant state rather than simply reflecting the intrinsic properties of tumor cells alone.

Cancer-associated fibroblasts (CAFs) are among the best-characterized stromal drivers of this process, although the strength of evidence differs between individual pathways. One study showed that CAF-derived exosomes enriched in the long noncoding RNA LINC00355 are taken up by BCa cells, where LINC00355 sponges miR-34b-5p, upregulates the drug efflux pump ABCB1, increases cisplatin export, and reduces intracellular drug accumulation. In T24 and 5637 cells, silencing LINC00355 or blocking CAF-Exo release restores cisplatin sensitivity, consistent with CAF-Exo acting as “molecular relay stations” that disseminate a resistant phenotype (Luo et al., 2021). However, these findings are largely confined to a small number of cell line models and lack independent validation in patient-derived samples, so it is not yet clear how broadly this mechanism operates across molecular subtypes. By contrast, CAF-derived CXCL14 appears to be supported by a broader experimental platform: CAFs from cisplatin-resistant patients secrete higher CXCL14, which binds CCR7 on BCa cells, activates STAT3, upregulates the NER factor ERCC4, and promotes cisplatin resistance *in vitro*, in ex vivo patient-derived organoids, and *in vivo* xenografts, where CCR7 or STAT3 inhibition reverses chemoresistance and enhances cisplatin-induced tumor cell death (Li et al., 2025b). In the same setting, STAT3 activation also induces glycolytic enzymes HK2 and LDHA, increases glycolytic flux, and drives normal fibroblasts toward a CXCL14-secreting CAF phenotype, establishing a self-reinforcing loop that couples DNA repair and metabolic rewiring. This multi-level evidence suggests that the CXCL14/CCR7/STAT3–ERCC4 axis represents a more mature stromal target than the LINC00355/miR-34b-5p/ABCB1 circuit, although prospective clinical testing is still lacking.

Tumor-associated macrophages (TAMs) provide a second stromal axis that converges on cisplatin handling, again with data that are convincing at the mechanistic level but still early in translational terms. Stimulation with TAM-conditioned medium induces marked upregulation of the detoxifying enzyme GSTO1 in BCa cells and triggers the release of large EVs that facilitate cisplatin efflux and reduce the formation of cisplatin–DNA adducts, thereby weakening the cytotoxic effect of the drug. Pharmacological inhibition of EV release counteracts GSTO1-associated resistance *in vitro* and in mouse models, suggesting that the TAM–GSTO1–EV axis offers a route for the TME to sequester and export cisplatin (Pan et al., 2024). Whether this mechanism is equally relevant in human tumors with more heterogeneous TAM phenotypes and variable prior treatments is still unknown.

Local physicochemical conditions also tune drug response. In an *in vitro* model, acidification of the culture medium to around pH 6.0 caused BCa cells to gradually acquire resistance, such that roughly double the cisplatin concentration was required to achieve comparable toxicity compared with pH 7.5, and this shift was accompanied by upregulation of the anti-apoptotic proteins Bcl-2 and XIAP and activation of LC3B-mediated autophagy, which together suppressed cisplatin-induced apoptosis and autophagic cell death (Hiruma et al., 2025). These data implicate acidosis as an active regulator of survival signaling rather than a neutral by-product of tumor metabolism, but so far evidence comes from controlled *in vitro* settings. Whether realistic pH gradients in human tumors reach the same thresholds and can be safely manipulated therapeutically remains to be addressed.

Remodeling of the immune microenvironment adds a further layer of complexity and raises questions about causality *versus* correlation. Analyses of public datasets and an internal cohort indicate that CA125-positive BCa cases have lower disease-free and overall survival than CA125-negative cases, often show mucin-rich regions surrounding tumor cells, and harbor an immunosuppressive milieu enriched in regulatory T cells and M2 macrophages, which associates with resistance to gemcitabine/cisplatin therapy (Yamashita et al., 2023). In non-muscle-invasive disease, citron kinase (CIT) has been identified as a biomarker of more aggressive tumors and poor prognosis: high CIT expression predicts inferior response to intravesical BCG and reduced sensitivity to commonly used chemotherapeutic agents, including adriamycin, gemcitabine, and cisplatin, and correlates with decreased intratumoral CD8^+^ T-cell infiltration (Deng et al., 2025b). These observations suggest that immunosuppression and effector T-cell dysfunction co-define a resistance-associated immune contexture, but it remains difficult to disentangle whether CA125 and CIT primarily act as upstream drivers of chemoresistance or as downstream surrogates of globally more aggressive biology and pre-existing immune exhaustion.

Taken together, current data support a model in which CAF-derived exosomes, TAM-driven GSTO1 induction, and immunosuppressive remodeling cooperate to establish a cisplatin-resistant niche. Most of the mechanistic insights are derived from cell lines and mouse models, yet several stromal pathways, most notably CXCL14/CCR7/STAT3–ERCC4 and CA125- or CIT-associated immune changes, already show associations with patient outcomes. Table 1 summarizes representative cell membrane- and TME-related regulators and their net effects on cisplatin sensitivity in bladder cancer.

**Table 1:** Key molecules and microenvironment-associated factors involved in cisplatin resistance in bladder cancer.

Factor	Cisplatin sensitivity	Reference
high OCT4-pg5 expression	↓	Zhou et al. (2024)
high ADNP expression	↓	Xie et al. (2021)
high PRDX2 expression	↓	Li et al. (2024c)
high TYMP expression	↓	Tian et al. (2025)
mitochondrial TACO1 translocation	↓	Deng et al. (2025a)
high TRPV4 expression	↓	Katari et al. (2025)
high CAF-derived CXCL14 expression	↓	Li et al. (2025b)
GSTO1-associated large extracellular vesicle release	↓	Pan et al. (2024)
CA125-associated tumor microenvironment	↓	Yamashita et al. (2023)
high CIT expression	↓	Deng et al. (2025b)

**Notes.**

↑ indicates increased cisplatin sensitivity (sensitizing association); ↓ indicates decreased cisplatin sensitivity (resistance-associated association). Unless otherwise specified, arrows refer to the effect associated with increased expression, activity, localization, or functional involvement of the listed factor.

## Cytoplasm

### Adaptive metabolic reprogramming

Adaptive metabolic reprogramming describes how tumor cells remodel metabolic pathways under tumor microenvironment (TME) pressure to maintain survival and proliferation (Otakhor & Soladoye, 2024). In BCa, this plasticity not only supports growth in energy- and nutrient-stressed conditions but also directly contributes to cisplatin resistance. Among the diverse changes reported, dysregulated glycolysis and lactate handling, lipid metabolism, amino acid and redox metabolism, and ferroptosis suppression emerge as four major axes that shape drug response.

At the glycolytic level, bladder cancer cells often show a persistent glycolytic bias even in the presence of adequate oxygen, consistent with the “Warburg effect” (Navarro et al., 2022). One study identified SRC hyperactivation as a central driver of resistance: SRC activates hexokinase 2, enhances glycolysis and the pentose phosphate pathway, increases nucleotide synthesis and NADPH generation, neutralizes cisplatin-induced reactive oxygen species (ROS), and protects tumor cells from DNA damage; genetic SRC ablation or pharmacological inhibition with eCF506 reverses this phenotype and restores cisplatin sensitivity *in vitro*, and eCF506 also enhances cisplatin efficacy in both CDX and PDX models derived from cisplatin-resistant patients (Gong et al., 2025). Metabolic profiling of matched parental and resistant cell lines further highlights that resistance is not restricted to a single “Warburg-like” pattern: in highly resistant HT1376R cells, glycolysis and proton efflux are increased and, over time, metabolism drifts toward lactate uptake, lipid biosynthesis, and glutamate metabolism, whereas KU1919R cells instead revert to greater dependence on oxidative phosphorylation (Afonso et al., 2024). Complementary transcriptomic work shows that lactylation-related genes define distinct bladder urothelial carcinoma (BLCA) clusters with different outcomes and immune microenvironment features; a lactylation-based risk score predicts prognosis and immunotherapy response, and DHCR7 emerges as a key marker whose knockdown enhances cisplatin and immunotherapy efficacy in experimental models (Zhao et al., 2025). Collectively, these studies support metabolic targeting of SRC or DHCR7 as promising strategies, but they also underline important limitations: the data rely on a restricted panel of cell lines and small PDX series, and intertumor heterogeneity suggests that individual resistant clones may occupy very different positions along the glycolysis–OXPHOS–lactate spectrum.

Lipid metabolism provides another layer of adaptation at the intersection of energy supply and membrane remodeling. MicroRNA profiling in cisplatin-resistant lines has identified EHHADH, a fatty acid β-oxidation enzyme, as a direct target of the tumor-suppressive miR-486-5p; miR-486-5p is downregulated in resistant cells, and its restoration inhibits proliferation, migration, and invasion and increases cisplatin sensitivity, whereas EHHADH knockdown phenocopies these effects, linking aberrant fatty acid catabolism to drug response (Okamura et al., 2021). In the cholesterol pathway, FDFT1 acts as a tumor suppressor and chemosensitivity regulator: its expression is reduced in resistant T24R cells, and loss of FDFT1 redirects flux from the sterol branch toward non-sterol isoprenoids, favoring prenylation of Ras and Rho family proteins that promote progression and chemoresistance; the miR-146b-5p/FDFT1 axis therefore emerges as a potential target to prevent this metabolic rerouting (Azhar et al., 2024). LRP8, a regulator of lipid uptake and storage, is upregulated in BCa tissues and cell lines, and its overexpression enhances proliferation, migration, invasion, phospholipid and triglyceride accumulation, and cisplatin resistance, whereas inhibition of lipid metabolism reverses these phenotypes (Wu et al., 2025a). Beyond endogenous regulators, diosgenin, a natural product from Dioscorea species, upregulates miR-195-5p and directly binds FASN, thereby modulating the FASN/SLC3A2 axis and enhancing cisplatin cytotoxicity in resistant BCa cells; at the same time, *in vivo* data reveal a paradoxical promotion of MNU-induced bladder tumorigenesis and renal toxicity, as well as cytotoxicity in normal urothelial and renal cells (Lai et al., 2025). Taken together, lipid-focused studies remain largely preclinical and sometimes yield conflicting safety signals, suggesting that fatty acid turnover, cholesterol routing, and lipid uptake are genuine modulators of resistance but that therapeutic manipulation, especially with agents such as diosgenin, will require careful dosing and patient selection to avoid worsening tumor behavior or off-target toxicity.

Amino acid and redox metabolism appear closely tied to both tumor stemness and the immune microenvironment. MAT2A, a key enzyme in methionine metabolism, is stabilized in cisplatin-resistant BCa cells and supports survival and stemness, whereas the circRNA circARHGAP10 normally promotes MAT2A degradation; in immunodeficient mouse models, circARHGAP10 overexpression combined with dietary methionine restriction reverses resistance, but the same regimen in immunocompetent hosts drives CD8^+^ T-cell exhaustion, and high expression of the amino acid transporter SLC7A6 correlates with low CD8^+^ T-cell infiltration and poor response, with co-inhibition of MAT2A and SLC7A6 needed to restore cisplatin sensitivity (Yang et al., 2023). Across multiple cohorts, dysregulated glutathione (GSH) dynamics represent another hallmark of NAC-resistant MIBC: GSH metabolism genes are enriched in resistant tumors, and GSH-handling proteins, including GLS1, associate with non-response; in cisplatin-resistant MIBC cells, pharmacological interference with GSH dynamics combined with cisplatin markedly suppresses tumor growth in orthotopic xenografts (Kim et al., 2023). Parallel analyses of mevalonate pathway activity using an MVAscore identify a high-mevalonate subtype (C1) characterized by increased pathway flux, advanced stage and grade, poor survival, reduced immune infiltration, and cisplatin resistance; mevalonate metabolism is also upregulated in resistant BCa cells, and simvastatin both inhibits proliferation and improves cisplatin sensitivity *in vitro* (Fang et al., 2025). These observations argue that amino acid and redox pathways offer attractive metabolic vulnerabilities, but they also caution that systemic interventions, such as methionine restriction or statin therapy, may have divergent effects on tumor cells *versus* immune compartments and therefore require validation in carefully designed *in vivo* and clinical studies.

Ferroptosis control adds a further critical dimension. Ferroptosis is a regulated form of cell death driven by iron-dependent lipid peroxidation, with GPX4 acting as a key antioxidant defense (Zhang et al., 2022). Cisplatin-resistant BCa cells not only evade cisplatin-induced death but can also show reduced sensitivity to GPX4 inhibitors (GPX4i); overexpression of TrxR1 underlies GPX4i resistance in these cells, and pharmacological TrxR1 inhibition with the natural diterpenoid Jolkinolide B restores GPX4i sensitivity and enhances anti-proliferative effects *in vitro* and *in vivo* (Yang et al., 2014). In parallel, SND1 binds the 3′UTR of GPX4 mRNA, stabilizes it, and maintains high GPX4 expression; SND1 knockdown promotes ferroptosis, increases ROS and iron accumulation, and overcomes cisplatin resistance, while GPX4 overexpression reverses these effects (Zhao et al., 2023). A distinct lncRNA-centered mechanism involves MAFG-AS1, which recruits the deubiquitinase UCHL5 to stabilize the iron chaperone PCBP2; PCBP2 then interacts with the iron exporter FPN1 to promote iron efflux and suppress ferroptosis, and MAFG-AS1 expression is transcriptionally driven by MAFG through an EP300/H3K27ac-dependent positive-feedback loop, with high MAFG-AS1 and MAFG levels correlating with advanced T and N stage and poor prognosis, and both serving as independent biomarkers of chemotherapy sensitivity in BCa (Xiang et al., 2021). Together, these ferroptosis-related studies suggest that resistant cells actively maintain ferroptosis-avoidance states through coordinated RNA–protein–iron-transport circuits, but most data still come from experimental systems and prognostic cohorts, and it remains unclear how broadly these mechanisms operate across molecular subtypes or under standard-of-care combination regimens.

Overall, metabolic reprogramming in cisplatin-resistant BCa operates through a web of glycolytic and lactylation changes, lipid pathway rewiring, methionine and GSH dynamics, mevalonate flux, and ferroptosis suppression. Many of these mechanisms are supported by *in vivo* models, PDXs, or patient-derived datasets and are linked to clinical features such as pathological stage, immune infiltration, and NAC response, yet almost all candidate targets remain at the preclinical stage. A critical next step will be to map how these metabolic circuits interface with EMT, CSC maintenance, and microenvironmental signals in longitudinal and treatment-stratified cohorts, to avoid merely shifting tumors to alternative metabolic states rather than truly dismantling the cisplatin-resistant phenotype.

### Activation of canonical signaling pathways

Classical oncogenic signaling pathways are a central layer of cisplatin resistance in BCa. Networks such as NF-κB, PI3K/AKT, and JAK2/STAT3 integrate survival cues and stress responses, and in doing so they influence DNA repair, apoptosis, and autophagy while reshaping cytoplasmic homeostasis to blunt the cytotoxic effects of cisplatin (Zhang et al., 2024; Lv, Zhao & Wu, 2025; Li et al., 2024a; Ding et al., 2021). In many experimental models, these pathways are not simply switched on in parallel, but are engaged by cisplatin itself and then sustained by transcriptional, epigenetic, or receptor-level feedback.

One of the clearest examples involves the EGFR–NF-κB axis. Cisplatin exposure robustly activates EGFR and downstream NF-κB, and high expression of the transcription factor TFAP2C maintains this activation over time, promoting survival and suppressing apoptosis. TFAP2C knockdown reduces EGFR/NF-κB signaling, induces cell cycle arrest, and enhances apoptotic death, which supports TFAP2C as a plausible sensitizing node rather than a passive marker of resistance (Xing et al., 2022). By contrast, ANXA6 emerges as a positive regulator of cisplatin response. Overexpression of ANXA6 enhances cisplatin-induced loss of viability, DNA damage, and apoptosis and can resensitize resistant cells; mechanistically, ANXA6 promotes formation of a PKCα/EGFR complex that restrains EGFR hyperphosphorylation and dampens downstream AKT and ERK1/2 activation, and its transcription is driven by PRRX1 (Cao et al., 2024a). Together, these data suggest that the balance between TFAP2C-driven NF-κB activation and PRRX1/ANXA6-mediated control of EGFR output helps decide whether EGFR signaling is channeled toward survival or toward chemosensitivity.

The PI3K/AKT pathway occupies a key position at the intersection of receptor feedback and RNA-based regulation. At the RNA-epigenetic level, the METTL3–YTHDF1 m6A machinery increases expression of the glycoprotein RPN2, which in turn activates PI3K/AKT/mTOR signaling and modulates proliferation and cisplatin response in BCa cells. In functional assays, restoring RPN2 can rescue the impaired growth and altered drug response caused by METTL3/YTHDF1 knockdown, indicating that this m6A–RPN2 axis is one of the routes through which PI3K/AKT activity is maintained (Zhu et al., 2023). At the receptor level, cisplatin upregulates the ErbB3 ligand heregulin-1β (HRG1/NRG1) and induces ErbB3 phosphorylation, which feeds into AKT and ERK activation; blocking HRG1–ErbB3 binding with the monoclonal antibody seribantumab suppresses this phosphorylation cascade and restores cisplatin sensitivity across both sensitive and resistant patient-derived xenograft models (Steele et al., 2023). Natural compounds can also tap into this circuitry. Saponins from Paris polyphylla directly inhibit the adaptor protein GRB2, disrupt the AKT–SREBP1 axis, and significantly resensitize BCa cells to cisplatin *in vitro* and in vivo; the key component polyphyllin VII has been identified as a GRB2-targeting molecule with translational potential (Liu et al., 2025a). Collectively, these results indicate that PI3K/AKT signaling in resistant BCa is sustained by a combination of m6A-dependent regulation, receptor-driven feedback, and scaffold proteins, and that several of these components (RPN2, ErbB3, and GRB2) have already entered the stage of preclinical pharmacological testing.

Aberrant JAK2/STAT3 signaling provides another recurrent theme linking invasion and chemoresistance. PAX2 upregulates ESPL1, activates JAK2/STAT3, and enhances survival and invasiveness of BCa cells, whereas silencing PAX2 or ESPL1 restores cisplatin sensitivity and improves responses when combined with cisplatin *in vivo* (Zhang et al., 2024). Independent work shows that cisplatin-resistant cells typically display high levels of phosphorylated STAT3 and that combining cisplatin with agents such as cyclanoline reduces p-STAT3, limits migration and invasion, and promotes apoptosis in cell culture and mouse models (Li et al., 2024b). In clinical cohorts, low expression of the chromatin remodeler SNF5 is associated with persistent STAT3 activation, more aggressive disease, and poor response to first-line cisplatin/gemcitabine; interestingly, these SNF5-deficient tumors appear more responsive to cisplatin combined with EGFR or EZH2 inhibitors, suggesting that SNF5 status may help stratify patients for specific STAT3-oriented combination regimens (Ding et al., 2021). Here, the evidence begins to bridge preclinical mechanistic work with biomarker-guided therapeutic decisions.

Taken together, current data support a model in which metabolic rewiring and aberrant activation of NF-κB, PI3K/AKT, and JAK2/STAT3 pathways cooperate at the cytoplasmic level to maintain a cisplatin-tolerant state. These signaling cascades are sustained through feedback involving membrane receptors, RNA modifications, and chromatin context, and in turn influence nuclear DNA damage responses and epigenetic remodeling. Table 2 collates key cytoplasmic and metabolic regulators discussed in this section and indicates whether they overall enhance or reduce cisplatin sensitivity.

**Table 2:** Intracellular regulators of cisplatin sensitivity in bladder cancer.

Factor	Cisplatin sensitivity	Reference
high SRC activity	↓	Gong et al. (2025)
high DHCR7 expression	↓	Zhao et al. (2025)
high EHHADH expression	↓	Okamura et al. (2021)
high FDFT1 expression	↑	Azhar et al. (2024)
high LRP8 expression	↓	Wu et al. (2025a)
high MAT2A expression	↓	Yang et al. (2023)
GLS1-associated GSH dynamics	↓	Kim et al. (2023)
high SND1 expression	↓	Zhao et al. (2023)
high ESPL1 expression	↓	Zhang et al. (2024)
high SNF5 expression	↑	Ding et al. (2021)
high TFAP2C expression	↓	Xing et al. (2022)
high ANXA6 expression	↑	Cao et al. (2024a)
high YTHDF1 expression	↓	Zhu et al. (2023)
high p-ErbB3	↓	Steele et al. (2023)

**Notes.**

↑ indicates increased cisplatin sensitivity (sensitizing association); ↓ indicates decreased cisplatin sensitivity (resistance-associated association). Unless otherwise specified, arrows refer to the effect associated with increased expression, activity, phosphorylation, or functional involvement of the listed factor. p-ErbB3 indicates phosphorylated ErbB3.

## Nucleus

### Enhanced DNA damage repair capacity

DNA damage response (DDR) is a core safeguard of genome stability, and its dysregulation is widely recognized as a major determinant of cisplatin resistance in BCa. Cisplatin generates intra- and inter-strand DNA crosslinks; if these lesions persist, cells undergo cell cycle arrest or apoptosis. Resistant cells, by contrast, upregulate specific DDR modules to contain cisplatin-induced damage and maintain proliferation under chronic drug pressure.

Within classical repair machineries, homologous recombination repair (HRR) is one of the best characterized routes to a resistant phenotype. MCM6 is highly expressed in cisplatin-treated BCa tissues with poor clinical response and promotes cell proliferation and invasion; knockdown of MCM6 enhances cisplatin sensitivity both *in vitro* and *in vivo*, and mechanistic work shows that MCM6 supports DDR while participating in a positive feedback loop with c-Myc, which transcriptionally activates MCM6 (Wang et al., 2025). The AP1M2/PUM1/RAD54B axis provides another way to sustain HRR under chemotherapy: AP1M2 is upregulated in gemcitabine/cisplatin-resistant cells and organoids, interacts with the RNA-binding protein PUM1, and stabilizes RAD54B mRNA, thereby maintaining high HR activity and tumor growth during treatment, whereas inhibition of this axis resensitizes xenografts to gemcitabine–cisplatin therapy (Liu et al., 2025b). In parallel, ZBTB11 is overexpressed in BCa, correlates with poor prognosis, and drives tumor growth; loss-of-function studies indicate that ZBTB11 transcriptionally regulates DDX1, promotes R-loop clearance, and limits DNA damage, and that the ZBTB11/DDX1 axis is required for BCa cells to tolerate cisplatin (Chen et al., 2022). Taken together, these studies support a cooperative HRR-promoting network that buffers cisplatin-induced lesions, but most data come from selected cell lines and preclinical models, so the relative contribution of each factor across molecular subtypes remains uncertain.

Aberrant activation of non-homologous end joining (NHEJ) offers an additional route to blunt cisplatin toxicity. circUGGT2 is upregulated in BCa, promotes migration and invasion, and associates with poor outcome; it binds the KU70/KU80 heterodimer and facilitates recruitment of NHEJ components such as DNA-PKcs and XRCC4 to chromatin after cisplatin treatment, thereby accelerating repair at DNA breaks and reducing drug efficacy (Lyu et al., 2024). IMP2 further strengthens this adaptation in muscle-invasive disease. It is overexpressed in chemoresistant MIBC and lung metastases, and its inhibition restores cisplatin sensitivity *in vitro* and in vivo; mechanistic work indicates that IMP2 stabilizes IPO4 and SLC7A11 mRNAs in an m6A-dependent manner, enhances nuclear translocation of C/EBPδ, activates PRKDC-mediated DNA repair, and simultaneously suppresses ferroptosis, so that DNA-damage resolution and death-pathway evasion are coupled within a single axis (Yan et al., 2024). HUS1, a component of the Rad9–Rad1–HUS1 clamp, is highly expressed in basal urothelial carcinoma cell lines, and high HUS1 levels correlate with worse survival in patients with BCa; knockdown of HUS1 suppresses proliferation of cisplatin-treated sensitive cells but does not fully resensitize established resistant lines, suggesting that HUS1 may be more informative as a prognostic marker than as a stand-alone therapeutic target in late-stage resistance (Lindner et al., 2022). These observations indicate that both HRR and NHEJ can be co-opted by BCa cells to tolerate cisplatin, with pathway dominance likely depending on genotype and disease stage.

Beyond these canonical routes, multiple RNA and chromatin modifiers modulate DDR capacity. NAT10-mediated ac4C RNA acetylation is induced by cisplatin and is associated with chemoresistance: NAT10 is upregulated in resistant BCa, driven by NF-κB p65 binding to its promoter, promotes DDR by binding and stabilizing AHNAK mRNA, and pharmacological inhibition of NAT10 with Remodelin resensitizes BCa organoids and xenografts to cisplatin (Xie et al., 2023). In contrast, the m6A reader YTHDC1 acts as a tumor suppressor in this setting. Reduced YTHDC1 expression destabilizes PTEN mRNA in an m6A-dependent manner, activates PI3K/AKT signaling, enhances DDR, and accelerates cell cycle recovery after damage; clinically, low YTHDC1 expression associates with poor response to cisplatin, whereas YTHDC1 overexpression restores sensitivity, and PI3K/AKT inhibition attenuates these effects (Su et al., 2023). Overall, these findings emphasize that RNA-modifying enzymes do not simply fine-tune gene expression but can directly set the threshold for DNA-damage tolerance, although evidence so far is largely limited to experimental models and small patient cohorts.

Nucleotide excision repair (NER) factors also shape cisplatin response. Large-scale clinical sequencing has consistently linked loss-of-function alterations in ERCC2 with increased cisplatin sensitivity and improved response to neoadjuvant chemotherapy in MIBC, and complementary preclinical work using macrophage-derived mimetic nanovesicles to co-deliver cisplatin and ERCC2 siRNA demonstrates that ERCC2 downregulation enhances DNA damage and apoptosis and suppresses tumor growth with limited off-target toxicity in MIBC models (Börcsök et al., 2025). Together, these data make ERCC2 one of the more robust predictive biomarkers for platinum benefit in BCa and suggest that it may also be a rational co-target in future combination strategies, though prospective validation is still lacking.

Noncoding RNAs introduce additional layers of regulation on top of these pathways. LINC02321 is one of six angiogenesis-related lncRNAs that form a prognostic signature in BCa and is the only member consistently upregulated in tumors; functional assays show that LINC02321 promotes proliferation, migration, invasion, and cisplatin resistance, likely *via* VEGFA-related signaling (Lu et al., 2024). At the level of cell cycle control, truncating mutations of CDKN1A (p21) show context-dependent effects. CRISPR-engineered early truncation leads to complete loss of p21, sustained DNA damage, defective cell cycle arrest, and increased cisplatin sensitivity, whereas clones expressing a C-terminally truncated p21 peptide display more efficient damage repair and enhanced resistance, in line with altered PCNA binding and nuclear localization (Shi et al., 2022; Sikder et al., 2021). These experiments illustrate that not all DDR-related alterations can be interpreted within a simple “more repair *versus* less repair” framework, and that the functional impact of p21 lesions depends on the exact truncation pattern and cellular context.

The phosphatase regulatory subunit PPP2R2B provides a further example of how nuclear transport and innate signaling intersect with DNA repair and drug response. PPP2R2B is downregulated in advanced BCa, and low expression promotes proliferation, migration, and cisplatin resistance. Mechanistically, PPP2R2B facilitates binding of IPO5 to ISG15 and promotes ISG15 nuclear translocation; nuclear ISG15 in turn inhibits DNA repair and activates the STING pathway, which further boosts ISG15 expression. When PPP2R2B is reduced, ISG15 nuclear entry is impaired, suppression of DNA repair is relieved, and cells become more repair-competent and drug-tolerant, while SUV39H1-mediated H3K9 trimethylation contributes to PPP2R2B downregulation and pharmacological inhibition of SUV39H1 can restore PPP2R2B levels (Huang et al., 2024b). This work suggests that epigenetic silencing of PPP2R2B removes a brake on repair capacity, but it also raises practical questions about how broadly STING–ISG15 signaling can be manipulated without undermining anti-tumor immunity.

Overall, enhanced DNA damage repair in cisplatin-resistant BCa does not arise from a single dominant lesion but from the combined action of HRR and NHEJ effectors, RNA and acylation readers, lncRNA–protein complexes, and cell cycle regulators. Some components, such as ERCC2 status, already provide clinically useful predictive information, whereas many others are still supported mainly by *in vitro* work and small preclinical models and will require testing in larger, treatment-annotated cohorts. This “repair-centered” adaptation interacts with the cytoplasmic signaling and metabolic networks described above, forming a multilayered barrier that limits the long-term efficacy of platinum-based chemotherapy.

### Epigenetic reprogramming

Epigenetic reprogramming is another major layer that supports cisplatin resistance in BCa. Rather than altering the underlying DNA sequence, resistant cells rewire gene expression programs through RNA regulation, chromatin remodeling, and DNA methylation. In this context, RNA modifications, noncoding RNAs, and chromatin/DNA changes form three interlocking mechanisms that collectively stabilize a drug-tolerant nuclear state.

At the level of RNA modification, N^6^-methyladenosine (m^6^A) remains the best-characterized mark. Loss of the SWI/SNF subunit ARID1A, a frequent event in BCa, drives upregulation of EIF4A3 and, in turn, circ0008399, a circRNA previously identified as an anti-apoptotic factor in cisplatin-treated cells; through this ARID1A–EIF4A3–circ0008399 axis, ARID1A-deficient cells attenuate cisplatin-induced apoptosis, and pharmacological inhibition of EIF4A3 reduces circ0008399 levels and resensitizes these cells to cisplatin, providing proof of concept that epitranscriptomic nodes can be druggable (Wei et al., 2021). A second m^6^A-centered pathway has been delineated around the E3 ligase RNF220: METTL3-mediated m^6^A marks on RNF220 transcripts are recognized by IGF2BP2, which stabilizes RNF220 mRNA and increases RNF220 protein abundance; RNF220 then ubiquitinates and destabilizes the pro-apoptotic factor PDE10A while at the same time promoting PD-L1 expression, thereby functionally coupling enhanced cisplatin resistance to immune escape (Li et al., 2025a). Beyond these specific axes, expression patterns of the demethylases ALKBH5 and FTO suggest that the “eraser” arm of the m^6^A machinery also modulates drug response, as both enzymes are downregulated in BLCA tissues and cell lines, and experimental knockdown further increases resistance to cisplatin and mitomycin C (Song et al., 2025). Overall, these m^6^A-focused studies link writers, readers, and erasers to drug resistance and immune modulation, although most of the mechanistic detail still comes from engineered cell lines and xenografts, and the stability of these signatures across diverse clinical scenarios remains uncertain.

Noncoding RNAs, particularly circRNAs, form a second epigenetic layer that is tightly intertwined with these RNA modifications. circ_104797 is markedly upregulated in cisplatin-resistant BCa cells and exhibits increased m^6^A levels that enhance its stability; functional work indicates that circ_104797 acts as a reservoir for miR-103a and miR-660-3p and maintains resistance, whereas targeted demethylation of circ_104797 promotes apoptosis under cisplatin exposure (Xu et al., 2024). Other circRNAs interface more directly with chromatin regulators. circSTX6 not only sponges miR-515-3p to relieve repression of SUZ12, a core PRC2 component, but also binds PABPC1 to stabilize SUZ12 mRNA, thereby reinforcing Polycomb-mediated silencing and enhancing resistance through a dual ceRNA–stabilization mechanism (Wei et al., 2024). At the level of transcription factors, BMI1 operates at the chromatin interface by increasing H2AK119 monoubiquitination at the miR-3682-3p promoter, suppressing this miRNA and consequently de-repressing the drug efflux transporter ABCB1; BMI1 overexpression enhances drug efflux and reduces cisplatin-induced apoptosis in dual cisplatin/gemcitabine-resistant models, whereas miR-3682-3p mimics or pharmacologic P-gp inhibition partially reverse this phenotype (Chen et al., 2021). Together, these studies illustrate how circRNAs and transcription factors converge on shared effector genes such as SUZ12 and ABCB1 and thus link RNA-level regulation to chromatin-embedded resistance programs, but they are largely based on a limited number of cell lines and patient samples, so it is not yet clear how generalizable these circuits are across molecular subtypes and treatment histories.

Chromatin and DNA-level modifications provide a third epigenetic axis that helps resistant cells maintain transcriptional homeostasis under chemotherapy-induced stress. SIRT7 and EZH2 act in concert at the promoter of RND3: SIRT7 reduces H3K18 acetylation, while EZH2 increases H3K27 trimethylation, and both changes suppress RND3 expression and reduce cisplatin sensitivity *in vitro* and in xenograft models; SIRT7 also decreases EZH2 succinylation, stabilizing EZH2 and further prolonging this repressive state (Cao et al., 2024b). In a complementary line of work, FOXC1 has been identified as a key transcription factor in “spontaneously” emerging invasive, drug-tolerant subpopulations that arise without deliberate drug selection. These cells display a reconfigured enhancer landscape enriched for FOXC1 motifs, and FOXC1 binding increases accessibility and upregulates neighboring genes such as ABCB1 and ID3, thereby promoting survival and facilitating a transition toward a cisplatin-resistant phenotype (Lu et al., 2022). A third example comes from the KEAP1–NRF2 axis. GULP1 normally binds KEAP1 and helps sequester NRF2 in the cytoplasm; promoter hypermethylation and silencing of GULP1 in BCa lead to constitutive NRF2 nuclear accumulation, upregulation of antioxidant genes such as HMOX1, and pronounced resistance to cisplatin *in vitro* and *in vivo*, while low GULP1 expression and GULP1 promoter methylation are enriched in muscle-invasive tumors and in non-responders to platinum-based chemotherapy (Hayashi et al., 2020). Compared with many of the earlier examples, GULP1 thus represents a relatively well-supported epigenetic lesion with both mechanistic and clinical correlations, although targeting NRF2 signaling in patients will need to balance tumor-specific effects against the risk of compromising cytoprotective responses in normal tissues.

Overall, epigenetic reprogramming in cisplatin-resistant BCa reflects a tightly coupled “repair–epigenetic” module: DDR factors provide the machinery to remove cisplatin-induced lesions, while RNA modifications, noncoding RNAs, and chromatin/DNA regulators determine which repair and survival genes remain active over time. The m^6^A machinery (METTL3–IGF2BP2–RNF220, ALKBH5/FTO), circRNA–Polycomb and BMI1–ABCB1 circuits, and chromatin regulators such as SIRT7/EZH2/FOXC1 and KEAP1–NRF2/GULP1 jointly stabilize the resistant state; at the same time, most of these targets are still supported predominantly by *in vitro* and early translational data, with only a few, such as GULP1 methylation, showing clear links to clinical response. Future work will need to test whether combining epigenetic modulators with platinum can effectively and safely disrupt these layered programs without simply driving the tumor toward alternative resistance routes. Table 3 provides an overview of nuclear factors, including DDR effectors, RNA modifiers, and chromatin regulators, that have been implicated in cisplatin response, together with their overall impact on drug sensitivity.

**Table 3:** Nucleus-associated mechanisms underpinning cisplatin resistance in bladder cancer.

Factor	Cisplatin sensitivity	Reference
high MCM6 expression	↓	Wang et al. (2025)
high AP1M2 expression	↓	Liu et al. (2025b)
high ZBTB11 expression	↓	Chen et al. (2022)
high IMP2 expression	↓	Yan et al. (2024)
high HUS1 expression	↓	Lindner et al. (2022)
high NAT10 expression	↓	Xie et al. (2023)
high YTHDC1 expression	↑	Su et al. (2023)
ERCC2 mutation/LOF	↑	Börcsök et al. (2025)
high HNRNPU expression	↓	Shi et al. (2022)
high PPP2R2B expression	↑	Huang et al. (2024b)
high RNF220 expression	↓	Li et al. (2025a)
high IGF2BP3 expression	↓	Song et al. (2025)
high ALKBH5 expression	↑	Drigeard Desgarnier et al. (2025)
high FTO expression	↑	Drigeard Desgarnier et al. (2025)
high SIRT7 expression	↓	Cao et al. (2024b)
high FOXC1 expression	↓	Lu et al. (2022)
high GULP1 expression	↑	Hayashi et al. (2020)

**Notes.**

↑ indicates increased cisplatin sensitivity (sensitizing association); ↓ indicates decreased cisplatin sensitivity (resistance-associated association). Unless otherwise specified, arrows refer to the effect associated with increased expression or activity of the listed factor, or with the specified molecular alteration when indicated (e.g., mutation/LOF).

## Organelles

### Inhibition of apoptosis

Apoptosis is a core safeguard that removes damaged or stressed cells, but in cisplatin-resistant bladder cancer this programmed death pathway is selectively attenuated, allowing tumor cells to persist under chemotherapy. Rather than being driven by a single lesion, resistant clones typically rewire apoptosis at multiple levels, from noncoding RNAs and viral factors to pro-survival signaling proteins and hormone receptors.

At the transcriptional and noncoding RNA level, loss of pro-apoptotic microRNA tone is a recurrent feature. Downregulation of miR-325 is consistently observed in resistant sublines such as 5637/R and T24/R; restoring its expression induces mitochondrial membrane depolarization and activates caspase-9, caspase-7, and caspase-3, thereby augmenting cisplatin cytotoxicity, whereas overexpression of the anti-apoptotic protein HAX-1 abolishes this sensitizing effect (Li, Zheng & Huang, 2020). Viral factors can also modulate apoptotic sensitivity. BK polyomavirus (BKPyV) infection increases the levels of viral miR-B1-5p, which directly targets ATF3, suppresses cisplatin-induced ATF3 upregulation, and reduces caspase-3 and PARP cleavage; forced expression of ATF3 in BKPyV-infected cells restores cisplatin-induced cytotoxicity and apoptosis, highlighting a BKPyV–miR-B1–ATF3 axis that promotes drug tolerance (Li et al., 2025d). Other noncoding RNAs tilt the balance toward apoptosis. circLIFR is frequently downregulated in MIBC and positively associated with favorable prognosis; mechanistically, it binds MSH2, enhances the MutSα–ATM interaction, stabilizes p73, and thereby facilitates cisplatin-induced apoptosis. In xenograft and PDX models, tumors with high circLIFR/MSH2 expression show superior responses to cisplatin-based chemotherapy (Zhang et al., 2021). In parallel, the novel glycoprotein SBSPON, often silenced in bladder cancer and linked to poor survival, acts as an HSPA5-binding tumor suppressor: by sequestering HSPA5 and preventing its membrane translocation, SBSPON inactivates AKT signaling, weakens HSPA5-mediated anti-apoptotic protection, and restores cisplatin sensitivity while limiting proliferation and migration *in vitro* and *in vivo* (Ni et al., 2025).

At the intracellular signaling level, several axes further consolidate this anti-apoptotic bias. Constitutive activation of NF-κB signaling is particularly prominent in claudin-low MIBC and correlates with poorer disease-specific survival; platinum-based chemotherapy further boosts NF-κB activity and induces classical target genes, including SPHK1, PLAUR, and SERPINE1, thereby reinforcing pro-survival and pro-invasive programs (Qin et al., 2021). Pharmacologic NF-κB inhibition with DMAPT in combination with cisplatin significantly improves antitumor efficacy in xenograft and immunocompetent mouse models while preserving body weight, renal function, and hematologic parameters, providing a strong preclinical rationale for NF-κB-centered chemosensitization strategies (Qin et al., 2021). Systemic endocrine and metabolic status also feeds into apoptotic control. In cisplatin-resistant patients and cell lines, vitamin D3 levels are reduced whereas SIRT1 is upregulated; vitamin D3 treatment suppresses SIRT1 expression, limits mitochondrial ROS-driven autophagy, and enhances cisplatin-induced apoptosis, while forced SIRT1 overexpression abolishes this resensitizing effect (Pan, Chen & Hu, 2022; Di et al., 2022). In addition, estrogen receptor-β (ERβ) signaling has emerged as an important determinant of chemoresponse. ERβ positivity is significantly enriched among non-responders to cisplatin–gemcitabine neoadjuvant chemotherapy, particularly in female patients, and ERβ expression is elevated in cisplatin-resistant sublines. ERβ antagonism with tamoxifen or upstream inhibition of the COX-2/EP2/EP4 axis reduces ERβ levels and partially restores cisplatin sensitivity, whereas 17β-estradiol enhances β-catenin activity and further promotes resistance (Li et al., 2025c).

Taken together, bladder cancer cells attenuate apoptotic signaling through a combination of miRNA/viral reprogramming, chaperone- and hormone-driven survival signaling, and NF-κB-centered stress adaptation, collectively securing a survival advantage under cisplatin treatment. Nevertheless, inhibition of apoptosis is only one pillar of the resistant phenotype; accumulating evidence indicates that hyperactivation of autophagy provides parallel energetic and homeostatic support, further expanding the survival niche of drug-tolerant clones and underscoring the need to consider multiple death and stress-response modalities in future combination strategies.

### Aberrant activation of autophagy

Autophagy, a key process for maintaining cellular homeostasis and metabolic balance, is frequently hyperactivated in cisplatin-resistant bladder cancer. CAB39 (calcium binding protein 39) is markedly upregulated in resistant cells, where it activates the LKB1–AMPK–LC3 (liver kinase B1/AMP-activated protein kinase/microtubule-associated protein 1 light chain 3) signaling axis to preserve mitochondrial function and lower intracellular ROS levels, thereby offsetting the cytotoxic effects of cisplatin. In animal models, either CAB39 knockdown or its combination with the autophagy inhibitor chloroquine (CQ) significantly restores drug sensitivity, underscoring its pivotal role in sustaining the resistant state (Gao et al., 2023). Another key regulator is the RNA-splicing factor SRSF1 (serine and arginine rich splicing factor 1), which promotes selective splicing of HIF1A (hypoxia-inducible factor-1α), upregulates BNIP3 (BCL2/adenovirus E1B 19 kDa interacting protein 3), and drives mitophagy, thereby dampening cisplatin responsiveness; clinically, high SRSF1 expression strongly correlates with poor prognosis (Wu et al., 2025b). Consistent with this, the natural lignan justicidin A has been shown to activate BNIP3-dependent mitophagy and markedly enhance cisplatin/gemcitabine sensitivity in HRAS-mutant bladder cancer cells, suggesting that pharmacologic manipulation of mitophagy may serve as a promising adjuvant chemotherapy strategy (Chang et al., 2025).

Beyond classical signaling cascades, epigenetic regulation also exerts profound control over autophagy-dependent resistance. The m6A methyltransferase METTL16 (methyltransferase-like protein 16) modulates autophagy by regulating the stability of PMEPA1 (prostate transmembrane protein, androgen induced 1) mRNA within the HIF-2α–METTL16–PMEPA1–autophagy axis: when highly expressed, METTL16 reduces PMEPA1 levels, suppresses autophagic flux, and enhances cisplatin sensitivity, whereas its downregulation promotes autophagy and favors resistance (Yu et al., 2024). In parallel, the deacetylase SIRT1 activates autophagy by deacetylating Beclin1 and thereby markedly reinforces the resistant phenotype; silencing SIRT1 or increasing Beclin1 acetylation effectively reverses this process and resensitizes tumor cells to cisplatin (Sun et al., 2024).

In summary, inhibition of apoptosis and abnormal activation of autophagic flux constitute complementary survival strategies at the organelle level, jointly sustaining cisplatin resistance in bladder cancer cells. At the organelle level, key apoptosis- and autophagy-related regulators discussed in ‘Inhibition of apoptosis’–‘Aberrant activation of autophagy’ are summarized in Table 4, together with their overall impact on cisplatin sensitivity. To facilitate cross-sectional comparison, Table 5 collates the noncoding RNAs mentioned throughout ‘Cell membrane’–‘Organelles’ and indicates their dominant functional modules (EMT/CSC, TME, metabolism, DDR, apoptosis) and net effect on cisplatin responsiveness.

**Table 4:** Organelle-associated factors modulating cisplatin resistance in bladder cancer.

Factor	Cisplatin sensitivity	Reference
high SPHK1 expression	↓	Qin et al. (2021)
PL-induced RIPK1 activation	↑	Pan, Chen & Hu (2022)
high SIRT1 expression	↓	Di et al. (2022), Sun et al. (2024)
high CAB39 expression	↓	Gao et al. (2023)
high SRSF1 expression	↓	Wu et al. (2025b)
high METTL16 expression	↑	Yu et al. (2024)

**Notes.**

↑ indicates increased cisplatin sensitivity (sensitizing association); ↓ indicates decreased cisplatin sensitivity (resistance-associated association). Unless otherwise specified, arrows refer to the effect associated with increased expression, activity, or functional involvement of the listed factor.

**Table 5:** Noncoding RNAs Involved in multilayered mechanisms of cisplatin resistance in bladder cancer.

Factor	Cisplatin sensitivity	Mechanism	Reference
high circATIC expression	↓	RCC2-mediated EMT	Huang et al. (2024a)
high HOXA-AS3 expression	↓	Notch1-mediated EMT	Chen et al. (2020)
high CAF-derived exosomal miR-146a-5p expression	↓	CSC/TME	Zhuang et al. (2023)
high CAF-derived exosomal LINC00355 expression	↓	miR-34b-5p/ABCB1/TME	Luo et al. (2021)
high MAFG-AS1 expression	↓	Ferroptosis inhibition	Xiang et al. (2021)
high miR-486-5p expression	↑	EHHADH-mediated lipid metabolism	Okamura et al. (2021)
high miR-146b-5p expression	↓	FDFT1-associated cholesterol metabolism	Azhar et al. (2024)
high circUGGT2 expression	↓	NHEJ-mediated DNA repair	Lyu et al. (2024)
high LINC02321 expression	↓	RUVBL2-mediated DNA repair	Lu et al. (2024)
high circ0008399 expression	↓	WTAP-mediated m6A methylation	Wei et al. (2021)
high circSTX6 expression	↓	miR-515-3p/PABPC1-SUZ12	Wei et al. (2024)
high circ_104797 expression	↓	m6A-dependent stabilization/ RNA sponge	Xu et al. (2024)
high miR-3682-3p expression	↑	ABCB1/P-glycoprotein-mediated drug efflux	Chen et al. (2021)
high miR-325 expression	↑	HAX-1-mediated apoptosis regulation	Li, Zheng & Huang (2020)
high miR-B1 expression	↓	ATF3-mediated apoptosis inhibition	Li et al. (2025d)
high circLIFR expression	↑	MutSα/ATM-p73-mediated apoptosis	Zhang et al. (2021)

**Notes.**

↑ indicates increased cisplatin sensitivity (sensitizing association); ↓ indicates decreased cisplatin sensitivity (resistance-associated association). Unless otherwise specified, arrows refer to the effect associated with increased expression or functional involvement of the listed factor.

## Integrated network model of cisplatin resistance in bladder cancer

**Figure 2:**
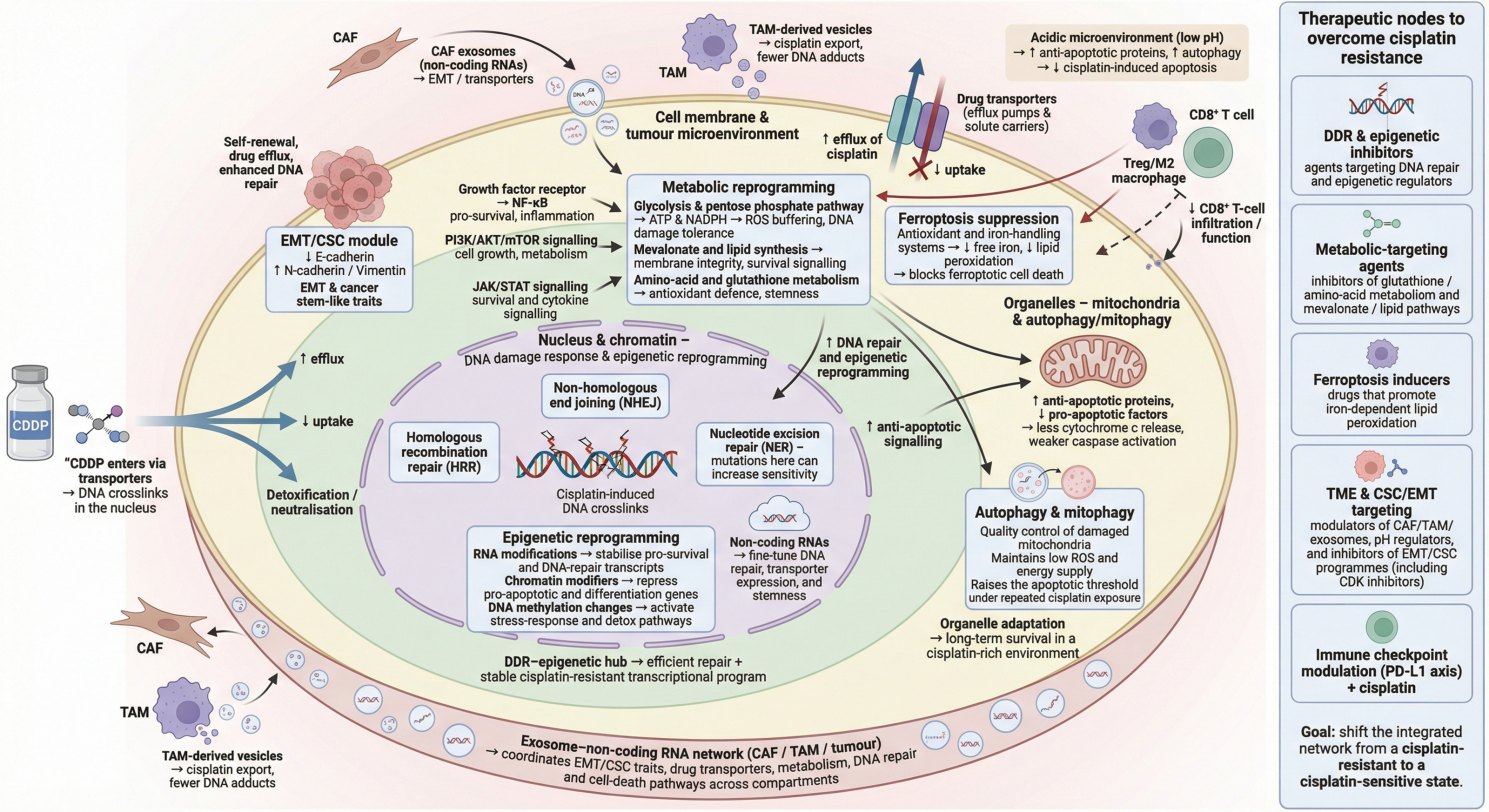
Multilayered, compartment-based overview of cisplatin resistance mechanisms in bladder cancer. Integrated network model of cisplatin resistance in bladder cancer, summarising how cell membrane/tumour microenvironment cues, cytoplasmic signalling and metabolism, nuclear DNA-damage/epigenetic programmes and organelle-centred pathways collectively shape cisplatin sensitivity.

The mechanisms described in the preceding sections suggest that cisplatin resistance in BCa is a network property (Fig. 2). Rather than arising within a single compartment, this resistant state depends on continuous crosstalk between the cell membrane and tumor microenvironment, the cytoplasm, the nucleus, and intracellular organelles.

At the cell membrane and within the tumor microenvironment, epithelial–mesenchymal transition (EMT), cancer stem cell-like traits, and shifts in stromal and immune cell populations reshape how tumor cells sense and process external cues. Changes in integrins, adhesion molecules, and receptors for growth factors or chemokines influence cell attachment and migration, but they also influence how transporters and channels are expressed and localized. In practical terms, these changes alter the amount of cisplatin that enters and leaves the cell and, therefore, the local drug concentration that reaches sensitive targets.

Membrane and microenvironmental signals are relayed into the cytoplasm, where they converge on a small number of stress and survival pathways, including PI3K/AKT, MAPK, and NF-κB signaling. These pathways organize adaptive metabolic programs. They shape glycolysis and oxidative phosphorylation and regulate glutathione and mevalonate metabolism, as well as key components of ferroptosis control. The resulting metabolic state sets the redox balance and influences membrane lipid composition. This metabolic profile then feeds back to the cell surface by modifying receptor clustering, lipid raft organization, and transporter function. In this way, signals at the membrane and processes in the cytoplasm form a two-way circuit that controls both cisplatin exposure and the capacity of cells to withstand cisplatin-induced oxidative and replicative stress.

Cytoplasmic signaling and metabolism are closely linked to nuclear responses. Stress-activated kinases and second messengers regulate DNA damage response (DDR) proteins, transcription factors, and chromatin-modifying enzymes. These nuclear changes determine how cisplatin-induced DNA lesions are recognized and repaired and whether cells arrest, undergo apoptosis, or remain in a tolerant state. In parallel, RNA- and chromatin-based epigenetic mechanisms adjust gene expression programs that concern EMT and stemness, transport systems, antioxidant defenses, apoptotic regulators, and autophagy-related proteins. The nucleus therefore functions as a central hub that receives upstream signals from the membrane and cytoplasm and translates them into transcriptional patterns that stabilize the resistant phenotype.

Organelle-centered processes add another level of regulation and feedback. Mitochondria integrate inputs from the cytoplasm and nucleus through changes in BCL-2 family proteins, p53 activity, and metabolic flux. These changes set the threshold for mitochondrial outer membrane permeabilization and caspase activation. Lysosome-dependent autophagy and mitophagy further adjust this threshold by removing damaged organelles and recycling nutrients. Cells that maintain this balance can survive repeated cycles of cisplatin exposure without triggering irreversible apoptosis. Stress signals that arise from dysfunctional mitochondria or lysosomes can activate nuclear transcriptional responses and cytoplasmic kinase pathways, closing additional feedback loops within the network.

Noncoding RNAs and exosome-mediated communication extend this network across the tumor as a whole. MicroRNAs, long noncoding RNAs, and circular RNAs regulate groups of targets in EMT pathways, transporter systems, DDR components, metabolic enzymes, and apoptotic machinery at the same time. Selective packaging of these RNAs into exosomes allows resistant cells to transfer their regulatory state to neighboring tumor cells and to stromal or immune cells. This cell-to-cell transfer helps to spread and stabilize cisplatin resistance within the tumor microenvironment.

These observations support a model in which cisplatin resistance in BCa is maintained by a limited number of cross-compartment regulatory axes. These axes connect membrane and microenvironmental cues, cytoplasmic metabolism and stress signaling, nuclear DDR and epigenetic control, and organelle-based regulation of cell death. From this perspective, it is unlikely that inhibition of a single downstream effector will provide durable resensitization to cisplatin. More promising approaches may combine cisplatin with agents that act on selected nodes in this network, such as DDR–epigenetic hubs, glutathione or mevalonate metabolism, ferroptosis-related circuits, or exosome–noncoding RNA signaling, with the aim of shifting the overall network from a resistant toward a cisplatin-sensitive state.

## Gaps and future directions

Despite rapid progress, several gaps remain. First, cross-layer causality remains insufficiently resolved: many nodes, such as EMT–CSC programs, ferroptosis control, and DDR–epigenetic coupling, are supported by correlative multi-omics evidence, but their causal wiring across cellular layers remains incompletely defined. Second, clinical validation is still limited: few mechanisms have undergone prospective, multicenter validation with standardized endpoints, such as pathologic response to platinum and survival, and with pre-specified biomarkers. Third, temporal dynamics remain poorly characterized: longitudinal sampling under cisplatin pressure, from pre-treatment to on-treatment and progression, is still scarce, limiting insight into evolutionary trajectories. Fourth, therapeutic translation remains challenging: rational combination strategies, such as ferroptosis induction plus DDR modulation or metabolic targeting plus immunomodulation, require pharmacodynamic biomarkers and drug–drug scheduling studies. Fifth, artificial intelligence integration remains underdeveloped: although digital pathology and imaging-based artificial intelligence can stratify risk, external generalizability, calibration, and fairness across centers and subgroups remain underreported. Addressing these gaps will enable biomarker-informed stratification and mechanism-anchored combination strategies to overcome cisplatin resistance.

## Conclusions

Cisplatin remains foundational in the management of muscle-invasive and metastatic bladder cancer, yet primary and acquired resistance continue to limit durable benefit. Such resistance is not attributable to a single pathway but reflects a systems-level adaptation involving drug handling and detoxification, DNA damage repair and fork homeostasis, rewired cell death programs, and modulation by the tumor microenvironment.

Framing the evidence across cellular layers, including the membrane, cytoplasm, nucleus/chromatin, and organelles, clarifies how EMT/CSC programs and stromal cues interface with transporters and ion channels; how metabolic reprogramming intertwines with ferroptosis antagonism; how reinforced DDR couples to epigenetic remodeling; and how lowered mitochondrial apoptotic priming coincides with activation of autophagy and mitophagy. These layers are integrated through conserved signaling hubs and exosome–noncoding RNA axes, yielding a coordinated, plastic resistance network.

At a macro level, cisplatin resistance in bladder cancer is best understood as layered, interconnected, and plastic. Recognizing this architecture delineates a targetable mechanistic landscape and offers principled directions for therapy, supporting biomarker-informed patient stratification and the rational design of combination approaches, thereby providing a clear conceptual scaffold for future mechanistic work and translational studies.

## List of Abbreviations

ABCB1: ATP-binding cassette subfamily B member 1
ADNP: Activity-dependent neuroprotective protein
AHNAK: AHNAK nucleoprotein
AKT (PKB): AKT serine/threonine kinase (protein kinase B)
ALKBH5: AlkB homolog 5, RNA demethylase
AMPK: AMP-activated protein kinase
ANXA6: Annexin A6
AP1M2: Adaptor-related protein complex 1 subunit mu 2
ATF3: Activating transcription factor 3
Bax: BCL2-associated X protein
BCa: Bladder cancer
BCG: Bacillus Calmette–Guérin
Bcl-2: B-cell lymphoma 2
BLCA: Bladder urothelial carcinoma
BMI1: BMI1 proto-oncogene, polycomb ring finger
BNIP3: BCL2/adenovirus E1B 19 kDa-interacting protein 3
CAB39: Calcium binding protein 39
CA125 (MUC16): Cancer antigen 125
CAF: Cancer-associated fibroblast
CAF-Exo: CAF-derived exosomes
CDK6: Cyclin-dependent kinase 6
CDKN1A (p21): Cyclin-dependent kinase inhibitor 1A
CEBPB: CCAAT enhancer binding protein beta
circRNA: circular RNA
CIT: Citron Rho-interacting kinase
CQ: Chloroquine
CSC: Cancer stem cell
CXCL14: C-X-C motif chemokine ligand 14
DDR: DNA damage response
DDX1: DEAD-box helicase 1
DHCR7: 7-dehydrocholesterol reductase
DNA-PKcs (PRKDC): DNA-dependent protein kinase catalytic subunit
ECM: Extracellular matrix
EGFR: Epidermal growth factor receptor
EMT: Epithelial–mesenchymal transition
ERBB2 (HER2): Erb-B2 receptor tyrosine kinase 2
ERBB3 (HER3): Erb-B2 receptor tyrosine kinase 3
ERCC2: Excision repair cross-complementation group 2
ERK: Extracellular signal-regulated kinase
ESPL1: Extra spindle pole bodies like 1 (Separase)
EVs: Extracellular vesicles
EZH2: Enhancer of zeste homolog 2
FASN: Fatty acid synthase
FDFT1: Farnesyl-diphosphate farnesyltransferase 1
FOXC1: Forkhead box C1
FTO: Fat mass and obesity-associated protein
GDF15: Growth/differentiation factor 15
GLS1: Glutaminase 1
GPX4: Glutathione peroxidase 4
GRB2: Growth factor receptor bound protein 2
GSH: Glutathione
GULP1: GULP PTB domain containing engulfment adaptor 1
GSTO1: Glutathione S-transferase omega 1
H2AK119: Histone H2A lysine 119 ubiquitination
HAX-1: HCLS1-associated protein X-1
HIF1A: Hypoxia-inducible factor 1-alpha
HNRNPU: Heterogeneous nuclear ribonucleoprotein U
HRG1 (NRG1): Neuregulin 1 (also known as heregulin 1)
HSP90β: Heat shock protein 90 beta
HSPA5: Heat shock protein family A (Hsp70) member 5
HUS1: HUS1 checkpoint clamp component
IGF2BP2/3: Insulin-like growth factor 2 mRNA-binding protein 2/3
IMP2: Insulin-like growth factor 2 mRNA-binding protein 2
IPO4: Importin 4
ISG15: Interferon-stimulated gene 15
JAK2: Janus kinase 2
JNK: c-Jun N-terminal kinase
KEAP1: Kelch-like ECH-associated protein 1
KU70/KU80 (XRCC6/5): X-ray repair cross-complementing protein 6/5
LC3B: Microtubule associated protein 1 light chain 3 beta
LKB1 (STK11): Liver kinase B1
lncRNA: long noncoding RNA
LRP8: LDL receptor related protein 8
MAT2A: Methionine adenosyltransferase 2A
MCM6: Minichromosome maintenance complex component 6
METTL3/14/16: Methyltransferase like 3/14/16
miRNA: microRNA
MSH2: MutS homolog 2
MVAscore: Mevalonate pathway activity score
N-cadherin (CDH2): Cadherin-2
NADPH: Nicotinamide adenine dinucleotide phosphate
NAT10: N-acetyltransferase 10
ncRNA: noncoding RNA
NER: Nucleotide excision repair
NF-κB: Nuclear factor kappa-light-chain-enhancer of activated B cells
NHEJ: Non-homologous end joining
Notch1: Notch receptor 1
NRF2: Nuclear factor erythroid 2–related factor 2
OCT4-pg5: OCT4 pseudogene 5
OCT4B: Octamer-binding transcription factor 4 isoform B
OXPHOS: Oxidative phosphorylation
PABPC1: Poly(A)-binding protein cytoplasmic 1
PARP: Poly(ADP-ribose) polymerase
PAX2: Paired box 2
p-ErbB3: phosphorylated ErbB3
PCBP2: Poly(rC)-binding protein 2
PDE10A: Phosphodiesterase 10A
PI3K: Phosphoinositide 3-kinase
PI3K–AKT signaling pathway: Phosphoinositide 3-kinase–protein kinase B signaling pathway
PI3K/AKT/mTOR: PI3K–AKT–mTOR signaling pathway
PKCα: Protein kinase C alpha
PMEPA1: Prostate transmembrane protein, androgen induced 1
PPP2R2B: Protein phosphatase 2 regulatory subunit B beta
PRDX2: Peroxiredoxin 2
PRRX1: Paired related homeobox 1
PTEN: Phosphatase and tensin homolog
PUM1: Pumilio RNA binding family member 1
RAD54B: DNA repair and recombination protein RAD54 homolog B
RCC2: Regulator of chromosome condensation 2
RIPK1: Receptor-interacting serine/threonine-protein kinase 1
RNF220: Ring finger protein 220
RND3: Rho family GTPase 3
RPN2: Ribophorin II
RUVBL2: RuvB-like AAA ATPase 2
SBSPON: Somatomedin B and thrombospondin type 1 domain containing
SIRT1/7: Sirtuin 1/7
SLC3A2: Solute carrier family 3 member 2
SLC7A6: Solute carrier family 7 member 6
SLC7A11: Solute carrier family 7 member 11
SNAIL: Snail family transcription factors
SNF5 (SMARCB1): SWI/SNF related matrix-associated actin-dependent regulator of chromatin subfamily B member 1
SND1: Staphylococcal nuclease and Tudor domain containing 1
SREBP1: Sterol regulatory element-binding protein 1
SRD5A3: Steroid 5 alpha-reductase 3
SRSF1: Serine/arginine-rich splicing factor 1
STAT3: Signal transducer and activator of transcription 3
SUZ12: SUZ12 polycomb repressive complex 2 subunit
SVEP1: Sushi, von Willebrand factor type A, EGF and pentraxin domain containing 1
TACO1: Translational activator of cytochrome c oxidase I
TAM: Tumor-associated macrophage
TFAP2C: Transcription factor AP-2 gamma
TME: Tumor microenvironment
TNFAIP3: TNF alpha induced protein 3
TRPV4: Transient receptor potential cation channel subfamily V member 4
TWIST: Twist family transcription factors
TYMP: Thymidine phosphorylase
Vimentin (VIM): Vimentin
WTAP: Wilms tumor 1-associated protein
XIAP: X-linked inhibitor of apoptosis protein
YTHDC1: YTH domain containing 1
YTHDF1: YTH N6-methyladenosine RNA binding protein 1
ZBTB11: Zinc finger and BTB domain containing 11
ZEB: Zinc finger E-box-binding homeobox

## Additional Information and Declarations

### Data Availability

The following information was supplied regarding data availability: This is a Literature Review.

## References

[ref-1] Afonso J, Barbosa-Matos C, Silvestre R, Pereira-Vieira J, Gonçalves SM, Mendes-Alves C, Parpot P, Pinto J, Â Carapito, Guedes de Pinho P, Santos L, Longatto-Filho A, Baltazar F (2024). Cisplatin-resistant urothelial bladder cancer cells undergo metabolic reprogramming beyond the Warburg effect. Cancers.

[ref-2] Azhar NA, Paramanantham Y, Nor WMFSBWM, Said NABM (2024). MicroRNA-146b-5p/FDFT1 mediates cisplatin sensitivity in bladder cancer by redirecting cholesterol biosynthesis to the non-sterol branch. International Journal of Biochemistry and Cell Biology.

[ref-3] Börcsök J, Gopaul D, Devesa-Serrano D, Mooser C, Jonsson N, Cagiada M, Stormoen DR, Ataya MN, Guercio BJ, Kaimakliotis HZ, Iyer G, Lindorff-Larsen K, Dyrskjøt L, Mouw KW, Szallasi Z, Sørensen CS (2025). Quantitative functional profiling of ERCC2 mutations deciphers cisplatin sensitivity in bladder cancer. Journal of Clinical Investigation.

[ref-4] Cao J, Chen S, Wang J, Fan X, Liu S, Li X, Yang L (2024a). Transcription factor PRRX1-activated ANXA6 facilitates EGFR-PKCα complex formation and enhances cisplatin sensitivity in bladder cancer. Life Sciences.

[ref-5] Cao Y, Wang S, Ma J, Long M, Ma X, Yang X, Ji Y, Tang X, Liu J, Lin C, Yang Y, Du P (2024b). Mechanistic insights into SIRT7 and EZH2 regulation of cisplatin resistance in bladder cancer cells. Cell Death & Disease.

[ref-6] Chang K-H, Chen H-C, Chen C-Y, Tsai S-P, Hsu M-Y, Wang P-Y, Wu S-Y, Su C-L (2025). Natural lignan justicidin A-induced mitophagy as a targetable niche in bladder cancer. Chemico-Biological Interactions.

[ref-7] Chen S-H, Chang J-Y (2019). New insights into mechanisms of cisplatin resistance: from tumor cell to microenvironment. International Journal of Molecular Sciences.

[ref-8] Chen L, Liu Z, Tang H, Zhou Z, Chen J, Ma Z, Deng M, Li X, Wu Y, Zheng L, Zhou L, Zheng X, Liu Z (2022). Knockdown of ZBTB11 impedes R-loop elimination and increases the sensitivity to cisplatin by inhibiting DDX1 transcription in bladder cancer. Cell Proliferation.

[ref-9] Chen D, Xie S, Wu Y, Cui Y, Cai Y, Lan L, Yang H, Chen J, Chen W (2020). Reduction of bladder cancer chemosensitivity induced by the effect of HOXA-AS3 as a ceRNA for miR-455-5p that upregulates Notch1. Frontiers in Oncology.

[ref-10] Chen M-K, Zhou J-H, Wang P, Ye Y-L, Liu Y, Zhou J-W, Chen Z-J, Yang J-K, Liao D-Y, Liang Z-J, Xie X, Zhou Q-Z, Xue K-Y, Guo W-B, Xia M, Bao J-M, Yang C, Duan H-F, Wang H-Y, Huang Z-P, Qin Z-K, Liu C-D (2021). BMI1 activates P-glycoprotein *via* transcription repression of miR-3682-3p and enhances chemoresistance of bladder cancer cell. Aging.

[ref-11] Chow P-M, Chang Y-W, Kuo K-L, Lin W-C, Liu S-H, Huang K-H (2021). CDK7 inhibition by THZ1 suppresses cancer stemness in both chemonaïve and chemoresistant urothelial carcinoma *via* the hedgehog signaling pathway. Cancer Letters.

[ref-12] Connors JS, Zhang L, Sasaki K, Di Nardo CD, Short NJ, Daver N, Borthakur G, Kadia TM, Kantarjian H, Jabbour E, Goodell MA, Garcia-Manero G, Takahashi K (2023). Protective role of oxaliplatin among platinum-based therapies in the development of therapy-related myeloid neoplasms. Blood.

[ref-13] Deng P, Zhong C, He P, Liu Q, Peng S, Liu R, Yu H, Li J, Wang Y, Yin W, Liu Y, Yang J, Zhong W, Lu J, Cai C (2025b). Uncovering citron kinase as a key biomarker for predicting outcomes and therapy efficacy in non-muscle-invasive bladder cancer. Gene.

[ref-14] Deng M, Zhou Z, Chen J, Li X, Liu Z, Ye J, Wei W, Wang N, Peng Y, Luo X, Jiang L, Zhou F, Zheng X, Liu Z (2025a). Enhanced oxidative phosphorylation driven by TACO1 mitochondrial translocation promotes stemness and cisplatin resistance in bladder cancer. Advanced Science.

[ref-15] Di Y-C, Zhang Z-H, Sun Y-P, Song J (2022). Vitamin D3 sensitizes resistant human bladder cancer cells to cisplatin by regulating Sirtuin 1 gene expression. European Review for Medical and Pharmacological Sciences.

[ref-16] Ding H, Huang Y, Shi J, Wang L, Liu S, Zhao B, Liu Y, Yang J, Chen Z (2021). Attenuated expression of SNF5 facilitates progression of bladder cancer *via* STAT3 activation. Cancer Cell International.

[ref-17] Drigeard Desgarnier M-C, Monshaugen I, Ougland R, Klungland A (2025). The 6-methyladenine erasers ALKBH5 and FTO influence chemotherapy efficiency in bladder cancer cell lines. Annals of Translational Medicine.

[ref-18] Fang Q, You C, Xiao X, Liu Y, Yang W, Li Q, Qing L, Dong Z (2025). Mevalonate metabolic reprogramming drives cisplatin resistance in bladder cancer: mechanisms and therapeutic targeting. Protein and Peptide Letters.

[ref-19] Fotopoulou E, Titilas I, Ronconi L (2022). Metallodrugs as anticancer chemotherapeutics and diagnostic agents: a critical patent review (2010–2020). Recent Patents on Anti-Cancer Drug Discovery.

[ref-20] Galanski MS (2006). Recent developments in the field of anticancer platinum complexes. Recent Patents on Anti-Cancer Drug Discovery.

[ref-21] Gao D, Wang R, Gong Y, Yu X, Niu Q, Yang E, Fan G, Ma J, Chen C, Tao Y, Lu J, Wang Z (2023). CAB39 promotes cisplatin resistance in bladder cancer *via* the LKB1-AMPK-LC3 pathway. Free Radical Biology and Medicine.

[ref-22] Ghandour R, Singla N, Lotan Y (2019). Treatment options and outcomes in nonmetastatic muscle invasive bladder cancer. Trends Cancer.

[ref-23] Gong Y, Gao D, Shi Y, Fan G, Yu X, Yang E, Cheng H, Tian J, Ding H, Liu S, Fu S, Tao Y, Shui Y, Cheng L, Li L, Wang Z (2025). SRC enhanced cisplatin resistance in bladder cancer by reprogramming glycolysis and pentose phosphate pathway. Communications Biology.

[ref-24] Hayashi M, Guida E, Inokawa Y, Goldberg R, Reis LO, Ooki A, Pilli M, Sadhukhan P, Woo J, Choi W, Izumchenko E, Gonzalez LM, Marchionni L, Zhavoronkov A, Brait M, Bivalacqua T, Baras A, Netto GJ, Koch W, Singh A, Hoque MO (2020). GULP1 regulates the NRF2-KEAP1 signaling axis in urothelial carcinoma. Science Signaling.

[ref-25] Hiruma K, Bilim V, Kazama A, Shirono Y, Murata M, Tomita Y (2025). Acidic microenvironment enhances cisplatin resistance in bladder cancer *via* Bcl-2 and XIAP. Current Issues in Molecular Biology.

[ref-26] Huang G, Liu J, Yu A, Luo C, Zhu J, Wang Y, Dai Z, Zhang L, Feng Z, Lu J, Dong Z, Luo J, Chen W, Chen Z (2024b). Nuclear translocation of ISG15 regulated by PPP2R2B inhibits cisplatin resistance of bladder cancer. Cellular and Molecular Life Science.

[ref-27] Huang C, Yang Y, Wang X, Chen S, Liu Z, Li Z, Tang X, Zhang Q (2024a). PTBP1-mediated biogenesis of circATIC promotes progression and cisplatin resistance of bladder cancer. International Journal of Biological Sciences.

[ref-28] Katari V, Dalal K, Kondapalli N, Paruchuri S, Nadiminty N, Thodeti CK (2025). Transient receptor potential vanilloid type 4 channels mediate bladder cancer cell proliferation, migration, and chemoresistance. Journal of Pharmacology and Experimental Therapeutics.

[ref-29] Kim Y, Ju H, Yoo S-Y, Jeong J, Heo J, Lee S, Park J-M, Yoon SY, Jeong SU, Lee J, Yun H, Ryu C-M, Lee J, Nam YJ, Kwon H, Son J, Jeong G, Oh J-H, Sung CO, Jeong EM, An J, Won S, Hong B, Lee JL, Cho YM, Shin D-M (2023). Glutathione dynamics is a potential predictive and therapeutic trait for neoadjuvant chemotherapy response in bladder cancer. Cell Reports Medicine.

[ref-30] Lai X, Li Q, Wu F, Lin J, Chen J, Zheng H, Guo L (2020). Epithelial-mesenchymal transition and metabolic switching in cancer: lessons from somatic cell reprogramming. Frontiers in Cell and Developmental Biology.

[ref-31] Lai Y, Peng Z, He Z, Lu Z, Yan S, Nie Q, Xiang Y (2025). Dioscin initiates dual roles in bladder cancer progression *via* miR-195-5p/FASN/SLC3A2 axis-mediated cell death mechanisms. Translational Oncology.

[ref-32] Li Y-J, Chen Y-H, Wang J-W, Wu H-H, Hsu H-H, Ho D-R, Yang C-W, Tian Y-C (2025d). Suppression of cisplatin induced ATF3 expression and apoptosis by BK polyomavirus and its encoded microRNA in bladder cancer cells. Biomedicine and Pharmacotherapy.

[ref-33] Li L, Li C, Miao F, Chen W, Kong X, Ye R, Wang F (2024b). Cyclanoline reverses cisplatin resistance in bladder cancer cells by inhibiting the JAK2/STAT3 pathway. Anti-Cancer Agents in Medicinal Chemistry.

[ref-34] Li K, Li Y, Zhang Y, Lv J, Zhao T, Dong Y, Liu F, Wang J, Wei Y, Zhu Q (2025a). N6-methyladenosine-modified RNF220 induces cisplatin resistance and immune escape *via* regulating PDE10A K48-linked ubiquitination in bladder cancer. Biochemical Pharmacology.

[ref-35] Li Y, Liu S, Zhou M, Zhao Z, Song D, Guo H, Yang R (2025c). A COX-2-targeted Platinum(IV) prodrug induces apoptosis and reduces inflammation in bladder cancer models. Pharmaceuticals.

[ref-36] Li H, Ma H, Ma J, Qin F, Fan S, Kong S, Zhao S, Ma J (2024a). Unveiling the role of RAC3 in the growth and invasion of cisplatin-resistant bladder cancer cells. Journal of Cellular and Molecular Medicine.

[ref-37] Li P, Wang W, Zhu B, Wang Y, Li J, Wang C, Wang C, Li Q (2024c). PRDX2 regulates stemness contributing to cisplatin resistance and metastasis in bladder cancer. Environmental Toxicology.

[ref-38] Li F, Zhang H, Wang Y, Yao Z, Xie K, Mo Q, Fan Q, Hou L, Deng F, Tan W (2022). FGFBP1 as a potential biomarker predicting bacillus Calmette–Guérin response in bladder cancer. Frontiers in Immunology.

[ref-39] Li R, Zheng J-Z, Huang X (2020). Suppression of HAX-1 induced by miR-325 resensitizes bladder cancer cells to cisplatin-induced apoptosis. European Review for Medical and Pharmacological Sciences.

[ref-40] Li T, Zhu K, Tong H, Sun Y, Zhu J, Qin Z, Chen J, Wu L, Zhang X, Wang A, Gou X, Yin H, He W (2025b). Cancer-associated fibroblast derived CXCL14 drives cisplatin chemoresistance by enhancing nucleotide excision repair in bladder cancer. Journal of Experimental & Clinical Cancer Research.

[ref-41] Lima De Oliveira J, Moré Milan T, Longo Bighetti-Trevisan R, Fernandes RR, Machado Leopoldino A, Oliveira De Almeida L (2023). Epithelial–mesenchymal transition and cancer stem cells: a route to acquired cisplatin resistance through epigenetics in HNSCC. Oral Diseases.

[ref-42] Lindner AK, Furlan T, Orme JJ, Tulchiner G, Staudacher N, D’Andrea D, Culig Z, Pichler R (2022). HUS1 as a potential therapeutic target in urothelial cancer. Journal of Clinical Medicine.

[ref-43] Liu Z, Jia B, Zhai Z, Wu F, Jia B, Yang Z, Zhang Y (2025b). AP1M2 drives gemcitabine-cisplatin chemoresistance by enhancing RAD54B-associated DNA repair in bladder cancer. FASEB Journal.

[ref-44] Liu J, Liu Y-X, Song Y-S, Liu C-K, Yu F-X, Liu G, Xu T-R, Sang J (2025a). Targeting GRB2-Akt-SREBP1 signaling axis by saponins from Paris polyphylla Smith var. yunnanensis (Franch.) Hand.-Mazz to overcome cisplatin resistance in bladder cancer. Journal of Ethnopharmacology.

[ref-45] Lu C, Gao H, Li H, Luo N, Fan S, Li X, Deng R, He D, Zhao H (2024). A novel LINC02321 promotes cell proliferation and decreases cisplatin sensitivity in bladder cancer by regulating RUVBL2. Translational Oncology.

[ref-46] Lu Y-T, Xu T, Iqbal M, Hsieh T-C, Luo Z, Liang G, Farnham PJ, Rhie SK, Goldkorn A (2022). FOXC1 binds enhancers and promotes cisplatin resistance in bladder cancer. Cancer.

[ref-47] Luo G, Zhang Y, Wu Z, Zhang L, Liang C, Chen X (2021). Exosomal LINC00355 derived from cancer-associated fibroblasts promotes bladder cancer cell resistance to cisplatin by regulating miR-34b-5p/ABCB1 axis. Acta Biochimica et Biophysica Sinica.

[ref-48] Lv Z, Zhao S, Wu H (2025). LIMA1 inhibits cisplatin resistance and malignant biological behavior of bladder cancer cells by suppressing the Wnt/β-catenin pathway. BMC Medical Genomics.

[ref-49] Lyu F, Huang S, Yan Z, He Q, Liu C, Cheng L, Cong Y, Chen K, Song Y, Xing Y (2024). CircUGGT2 facilitates progression and cisplatin resistance of bladder cancer through nonhomologous end-joining pathway. Cellular Signalling.

[ref-50] Navarro C, Ortega Á, Santeliz R, Garrido B, Chacín M, Galban N, Vera I, De Sanctis JB, Bermúdez V (2022). Metabolic reprogramming in cancer cells: emerging molecular mechanisms and novel therapeutic approaches. Pharmaceutics.

[ref-51] Ni B, Li S, Zhao L, Gao L, Luo L, Zhang J, Xie X, Zhu Y, Yang W, Min S, Wang Y, Li X, Cai Z, Speakman JR, Li Z (2025). Novel glycoprotein SBSPON suppressed bladder cancer through the AKT signal pathway by inhibiting HSPA5 membrane translocation. International Journal of Biological Sciences.

[ref-52] Okamura S, Yoshino H, Kuroshima K, Tsuruda M, Osako Y, Sakaguchi T, Yonemori M, Yamada Y, Tatarano S, Nakagawa M, Enokida H (2021). EHHADH contributes to cisplatin resistance through regulation by tumor-suppressive microRNAs in bladder cancer. BMC Cancer.

[ref-53] Otakhor KO, Soladoye EO (2024). A review of metabolic reprogramming in cancer cells: mechanisms and therapeutic targets. World Journal of Advanced Research and Reviews.

[ref-54] Pan X, Chen G, Hu W (2022). Piperlongumine increases the sensitivity of bladder cancer to cisplatin by mitochondrial ROS. Journal of Clinical Laboratory Analysis.

[ref-55] Pan Y-C, Chu P-Y, Lin C-C, Hsieh C-Y, Hsu W-Y, Shyur L-F, Yang J-C, Chang W-C, Wu Y-C (2024). Glutathione S-transferase omega class 1 (GSTO1)-associated large extracellular vesicles are involved in tumor-associated macrophage-mediated cisplatin resistance in bladder cancer. Molecular Oncology.

[ref-56] Pedersen GL, Erikson MS, Mogensen K, Rosthøj S, Hermann GG (2023). Outpatient photodynamic diagnosis–guided laser destruction of bladder tumors is as good as conventional inpatient photodynamic diagnosis–guided transurethral tumor resection in patients with recurrent intermediate-risk low-grade Ta bladder tumors. A prospective randomized noninferiority clinical trial. European Urology.

[ref-57] Qin Z, Tong H, Li T, Cao H, Zhu J, Yin S, He W (2021). SPHK1 contributes to cisplatin resistance in bladder cancer cells *via* the NONO/STAT3 axis. International Journal of Molecular Medicine.

[ref-58] Shi Z-D, Hao L, Han X-X, Wu Z-X, Pang K, Dong Y, Qin J-X, Wang G-Y, Zhang X-M, Xia T, Liang Q, Zhao Y, Li R, Zhang S-Q, Zhang J-H, Chen J-G, Wang G-C, Chen Z-S, Han C-H (2022). Targeting HNRNPU to overcome cisplatin resistance in bladder cancer. Molecular Cancer.

[ref-59] Sikder RK, Ellithi M, Uzzo RN, Weader DJ, Metz AL, Behbahani A, McKenzie ER, El-Deiry WS, Abbosh PH (2021). Differential effects of clinically relevant N-*versus* C-terminal truncating CDKN1A mutations on cisplatin sensitivity in bladder cancer. Molecular Cancer Research.

[ref-60] Song Q, Wang W, Yu H, Zhou Z, Zhuang J, Lv J, Jiang L, Yang X, Lu Q, Yang H (2025). IGF2BP3 promotes the proliferation and cisplatin resistance of bladder cancer by enhancing the mRNA stability of CDK6 in an m6A dependent manner. International Journal of Biological Sciences.

[ref-61] Steele TM, Tsamouri MM, Siddiqui S, Lucchesi CA, Vasilatis D, Mooso BA, Durbin-Johnson BP, Ma A-H, Hejazi N, Parikh M, Mudryj M, Pan C-X, Ghosh PM (2023). Cisplatin-induced increase in heregulin 1 and its attenuation by the monoclonal ErbB3 antibody seribantumab in bladder cancer. Scientific Reports.

[ref-62] Su Y, Wang B, Huang J, Huang M, Lin T (2023). YTHDC1 positively regulates PTEN expression and plays a critical role in cisplatin resistance of bladder cancer. Cell Proliferation.

[ref-63] Sun Y, Liu X, Tong H, Yin H, Li T, Zhu J, Chen J, Wu L, Zhang X, Gou X, He W (2024). SIRT1 promotes cisplatin resistance in bladder cancer *via* Beclin1 deacetylation-mediated autophagy. Cancer.

[ref-64] Sung H, Ferlay J, Siegel RL, Laversanne M, Soerjomataram I, Jemal A, Bray F (2021). Global cancer statistics 2020: GLOBOCAN estimates of incidence and mortality worldwide for 36 cancers in 185 countries. CA: A Cancer Journal for Clinicians.

[ref-65] Tian S, Wang C, Zhao X, Xuan Y, Wei W, Dong Y, Tao W, Zhang C, Cai T, Liu C, Huang Y, Zhang X (2025). A CEBPB/TYMP/GDF15 signaling axis mediates tumor growth and cisplatin resistance in bladder cancer. Translational Oncology.

[ref-66] Wang J, Li X, Zhao L, Fan X, Cao J, Wang S, Li K, Wang H, Zhang Y, Wang H, Xu C, Ding L, Che T, Chen S, Yang L (2025). The MCM6-c-Myc positive feedback loop mediates bladder cancer progression and cisplatin resistance. International Journal of Biological Macromolecules.

[ref-67] Wei W, Liu K, Huang X, Tian S, Wang H, Zhang C, Ye J, Dong Y, An Z, Ma X, Wang B, Huang Y, Zhang X (2024). EIF4A3-mediated biogenesis of circSTX6 promotes bladder cancer metastasis and cisplatin resistance. Journal of Experimental & Clinical Cancer Research.

[ref-68] Wei W, Sun J, Zhang H, Xiao X, Huang C, Wang L, Zhong H, Jiang Y, Zhang X, Jiang G (2021). Circ0008399 interaction with WTAP promotes assembly and activity of the m6A methyltransferase complex and promotes cisplatin resistance in bladder cancer. Cancer Research.

[ref-69] Wu G-F, Luo Z-G, Gao K, Ren Y, Shen C, Ying X-R (2025a). LRP8 regulates lipid metabolism to stimulate malignant progression and cisplatin resistance in bladder cancer. The Kaohsiung Journal of Medical Sciences.

[ref-70] Wu Q, Yu H, Sun H, Lv J, Zhuang J, Cai L, Jiang L, Chen Y, Tao Y, Bai K, Yang H, Yang X, Lu Q (2025b). SRSF1-mediated alternative splicing regulates bladder cancer progression and cisplatin sensitivity through HIF1A/BNIP3/mitophagy axis. Journal of Translational Medicine.

[ref-71] Xiang L, Zeng Q, Liu J, Xiao M, He D, Zhang Q, Xie D, Deng M, Zhu Y, Liu Y, Bo H, Liu X, Zhou M, Xiong W, Zhou Y, Zhou J, Li X, Cao K (2021). MAFG-AS1/MAFG positive feedback loop contributes to cisplatin resistance in bladder urothelial carcinoma through antagonistic ferroptosis. Science Bulletin.

[ref-72] Xie R, Cheng L, Huang M, Huang L, Chen Z, Zhang Q, Li H, Lu J, Wang H, Zhou Q, Huang J, Chen X, Lin T (2023). NAT10 drives cisplatin chemoresistance by enhancing ac4C-associated DNA repair in bladder cancer. Cancer Research.

[ref-73] Xie Y, Zhu S, Zang J, Wu G, Wen Y, Liang Y, Long Y, Guo W, Zang C, Hu X, Fan G, Xiang S, Zhang J (2021). ADNP prompts the cisplatin-resistance of bladder cancer *via* TGF-β-mediated epithelial-mesenchymal transition (EMT) pathway. Journal of Cancer.

[ref-74] Xing J, Chen W, Chen K, Zhu S, Lin F, Qi Y, Zhang Y, Han S, Rao T, Ruan Y, Zhao S, Yu W, Cheng F (2022). TFAP2C knockdown sensitizes bladder cancer cells to cisplatin treatment *via* regulation of EGFR and NF-κB. Cancer.

[ref-75] Xu C, Zhou J, Zhang X, Kang X, Liu S, Song M, Chang C, Lin Y, Wang Y (2024). N6-methyladenosine-modified circ_104797 sustains cisplatin resistance in bladder cancer through acting as RNA sponges. Cellular & Molecular Biology Letters.

[ref-76] Yamashita T, Higashi M, Sugiyama H, Morozumi M, Momose S, Tamaru J-I (2023). Cancer antigen 125 expression enhances the gemcitabine/cisplatin-resistant tumor microenvironment in bladder cancer. American Journal of Pathology.

[ref-77] Yan Y, Huang Z, Zhu Z, Wang Y, Cao X, Yang C, Jiang J, Xia S, Shen B (2024). IMP2 drives chemoresistance by repressing cisplatin-induced apoptosis and ferroptosis *via* activation of IPO4 and SLC7A11 under hypoxia in bladder cancer. Cancer Cell International.

[ref-78] Yang C, Ou Y, Zhou Q, Liang Y, Li W, Chen Y, Chen W, Wu S, Chen Y, Dai X, Chen X, Chen T, Jin S, Liu Y, Zhang L, Liu S, Hu Y, Zou L, Mao S, Jiang H (2023). Methionine orchestrates the metabolism vulnerability in cisplatin resistant bladder cancer microenvironment. Cell Death & Disease.

[ref-79] Yang WS, SriRamaratnam R, Welsch ME, Shimada K, Skouta R, Viswanathan VS, Cheah JH, Clemons PA, Shamji AF, Clish CB, Brown LM, Girotti AW, Cornish VW, Schreiber SL, Stockwell BR (2014). Regulation of ferroptotic cancer cell death by GPX4. Cell.

[ref-80] Yu H, Zhuang J, Zhou Z, Song Q, Lv J, Yang X, Yang H, Lu Q (2024). METTL16 suppressed the proliferation and cisplatin-chemoresistance of bladder cancer by degrading PMEPA1 mRNA in a m6A manner through autophagy pathway. International Journal of Biological Sciences.

[ref-81] Zhang C, Liu X, Jin S, Chen Y, Guo R (2022). Ferroptosis in cancer therapy: a novel approach to reversing drug resistance. Molecular Cancer.

[ref-82] Zhang W, Wang Y, Tang Q, Li Z, Sun J, Zhao Z, Jiao D (2024). PAX2 mediated upregulation of ESPL1 contributes to cisplatin resistance in bladder cancer through activating the JAK2/STAT3 pathway. Naunyn-Schmiedebergs Archives of Pharmacology.

[ref-83] Zhang Y, Wang Z, Yu J, Shi JZ, Wang C, Fu WH, Chen ZW, Yang J (2012). Cancer stem-like cells contribute to cisplatin resistance and progression in bladder cancer. Cancer Letters.

[ref-84] Zhang H, Xiao X, Wei W, Huang C, Wang M, Wang L, He Y, Sun J, Jiang Y, Jiang G, Zhang X (2021). CircLIFR synergizes with MSH2 to attenuate chemoresistance *via* MutSα/ATM-p73 axis in bladder cancer. Molecular Cancer.

[ref-85] Zhao Y, Ren P, Yang Z, Wang L, Hu C (2023). Inhibition of SND1 overcomes chemoresistance in bladder cancer cells by promoting ferroptosis. Oncology Reports.

[ref-86] Zhao Y, Xing Z, Zhao Y, Xu H, Liu R, Yang T, Wang Y, Zhu X (2025). Lactylation prognostic signature identifies DHCR7 as a modulator of chemoresistance and immunotherapy efficacy in bladder cancer. Frontiers in Immunology.

[ref-87] Zhou W, Yang Y, Wang W, Yang C, Cao Z, Lin X, Zhang H, Xiao Y, Zhang X (2024). Pseudogene OCT4-pg5 upregulates OCT4B expression to promote bladder cancer progression by competing with miR-145-5p. Cell Cycle.

[ref-88] Zhu J, Tong H, Sun Y, Li T, Yang G, He W (2023). YTHDF1 promotes bladder cancer cell proliferation *via* the METTL3/YTHDF1-RPN2-PI3K/AKT/mTOR axis. International Journal of Molecular Sciences.

[ref-89] Zhuang J, Shen L, Li M, Sun J, Hao J, Li J, Zhu Z, Ge S, Zhang D, Guo H, Huang R, Yan J (2023). Cancer-associated fibroblast-derived miR-146a-5p generates a niche that promotes bladder cancer stemness and chemoresistance. Cancer Research.

